# Evidence for model-based encoding of Pavlovian contingencies in the human brain

**DOI:** 10.1038/s41467-019-08922-7

**Published:** 2019-03-07

**Authors:** Wolfgang M. Pauli, Giovanni Gentile, Sven Collette, Julian M. Tyszka, John P. O’Doherty

**Affiliations:** 1 Division of Humanities and Social Sciences, MC 228-77, California Institute of Technology, 1200 E. California Blvd, Pasadena, CA 91125 USA; 2 Computation and Neural Systems Program, MC 228-77, California Institute of Technology, 1200 E. California Blvd, Pasadena, CA 91125 USA; 3 Artificial Intelligence Platform, Microsoft, One Microsoft Way, Redmond, WA 98052 USA

## Abstract

Prominent accounts of Pavlovian conditioning successfully approximate the frequency and intensity of conditioned responses under the assumption that learning is exclusively model-free; that animals do not develop a cognitive map of events. However, these model-free approximations fall short of comprehensively capturing learning and behavior in Pavlovian conditioning. We therefore performed multivoxel pattern analysis of high-resolution functional MRI data in human participants to test for the encoding of stimulus-stimulus associations that could support model-based computations during Pavlovian conditioning. We found that dissociable sub-regions of the striatum encode predictions of stimulus-stimulus associations and predictive value, in a manner that is directly related to learning performance. Activity patterns in the orbitofrontal cortex were also found to be related to stimulus-stimulus as well as value encoding. These results suggest that the brain encodes model-based representations during Pavlovian conditioning, and that these representations are utilized in the service of behavior.

## Abstract (web summary)

Pavlovian conditioning involves model-free learning that associates predictive stimuli with their outcome value. Here, the authors present evidence for activation of OFC and striatum that is consistent with model based information during a pavlovian task with multiple stimuli that predict rewards.

## Introduction

An accumulating literature suggests that instrumental behavior is composed of two distinct computational mechanisms^1^. Behavior can be model-free (MF), because its execution has been consistently reinforced, resulting in the development of stimulus-response (SR) associations, as described by the law of effect^2^. Model-based behavior (MB) is thought to involve a cognitive model of environmental contingencies, and deliberation over potential actions based on anticipated outcomes^3^.

In contrast to instrumental conditioning, computational models of Pavlovian conditioning have tended to assume exclusively MF mechanisms. Such models typically implement incremental changes in predictions for conditioned stimuli (CS) proportional to how surprised the agent is by unconditioned stimuli (US)^4^. The most common implementation of this surprise signal in neuroscience is the signed reward prediction error (RPE) from the temporal difference (TD) algorithm^5^. TD learning assigns a scalar value to the CS, corresponding to the amount of reward available in the present state, plus discounted future rewards. It does not learn where a reward will be delivered, nor the sequence of events that led up to reward delivery. TD learning has gained strong empirical support because RPEs correlate with dopaminergic midbrain activity^6,7^, as well as BOLD responses in the ventral striatum^8^, which receives strong dopaminergic projections^9^.

However, MF algorithms fall short of comprehensively capturing learning and behavior during Pavlovian conditioning^10^. For instance, many Pavlovian conditioned responses are devaluation sensitive, in that the strength of a conditioned response to the CS is modulated by changes in the current value of the US, induced for instance by outcome devaluation^11^. Yet, according to MF algorithms, conditioned responses should be insensitive to immediate changes in US value, and would instead manifest only incremental changes as a function of re-learning. Other well-established conditioning phenomena such as sensory preconditioning^12^, are also not explicable by conventional MF learning.

Thus, behavioral evidence supports the likely recruitment of MB mechanisms during Pavlovian conditioning. Accordingly, Pavlovian conditioning might involve the encoding of stimulus–stimulus relationships, independently of their value, such that the presence of one stimulus can elicit a representation of the identity of an associated stimulus. In Pavlovian conditioning a cognitive map of sorts may be formed, whereby knowledge of the relationship between stimuli is encoded, allowing flexible computation of the associated value of those stimuli.

In spite of behavioral evidence for MB computations during Pavlovian conditioning, evidence for such computations in the brain remains sparse. Rodent studies reported evidence for MB knowledge in both orbitofrontal cortex (OFC) value signals and dopaminergic RPE signals^13–15^. Prévost et al.^16^ reported amygdala activity associated with the encoding of knowledge of the structure in a reversal-learning Pavlovian paradigm. Others have found unconditioned stimulus identity representations in the OFC^17,18^.

**Fig. 1:**
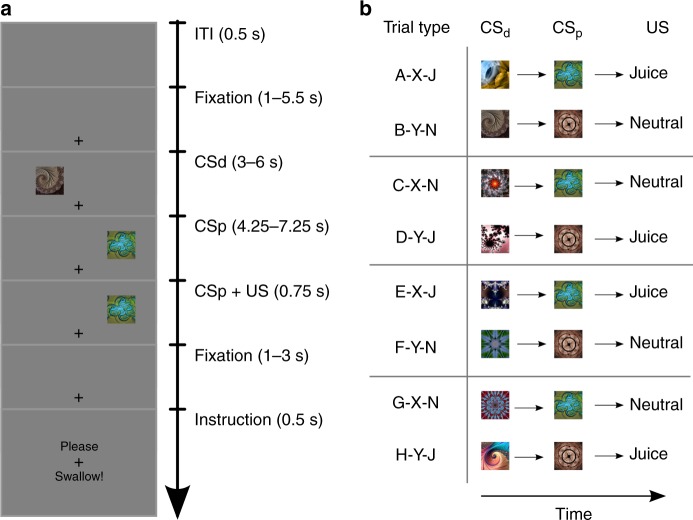
Sequential Pavlovian conditioning paradigm. **a** Each trial was initiated by the onset of a central fixation cross. The distal and CSp fractals were presented sequentially in two random locations on the screen. The presentation of the CSp co-terminated with US delivery. **b** Main trial types and stimulus categories in the 4 learning sessions. Identical CSp fractals (CS-X and CS-Y) were used throughout sessions, with valence reversals between sessions. Two unique CSd fractals were introduced in each session. ITI inter-trial interval

An open question remains: How does the brain encode the cognitive map, or state-space transition model, needed for MB value computations during Pavlovian conditioning? To address this question, we optimized a sequential Pavlovian conditioning paradigm^19^ for multivoxel pattern analyses (MVPA), and scanned human volunteers with functional MRI (fMRI) while they performed this task. On each trial, participants first encountered one of two visual distal conditioned stimulus (CSd) fractals, followed by one of two proximal conditioned stimulus (CSp) fractals, followed by the delivery of either an affectively pleasant or affectively neutral liquid (US) (Fig. 1). Each participant experienced 4 learning sessions, with 30 trials each. Before the first and after the last training session, participants rated the subjective value of CS fractals, as well as how hungry or thirsty they felt. Before concluding the study, participants reported their explicit knowledge of the Pavlovian associations. Specific associations between CSp fractals and the USs were reversed across sessions, while we selected novel CSd fractals for each session, so as to enable two independent MVPA classifiers to solve two orthogonal classification tasks regarding the CS fractals.

One classifier was trained on the value of CSp fractals, to then test its ability to predict the value of CSp fractals (in held-out learning sessions) based on the fMRI blood oxygenation level dependent (BOLD) response to the earlier CSd fractal. Critically, the results of this decoding analysis are independent of the visual features of CS fractals eliciting a BOLD response^17^. The ability to successfully predict the value of CSp fractals would be evidence either for anticipatory value representations of CSd fractals, or for anticipatory identity or value representations of the US. That is, successful predictions would be consistent with both MB and MF learning during Pavlovian conditioning. We trained a second classifier to decode the identity of CSp fractals, to test its ability to predict the identity of CSp fractals, again based on the BOLD response to the CSd fractal. Even though we anticipate that the CSp fractals will acquire affective value through learning, to correctly identify the identity of the CSp fractal, affective information has to be ignored by the classifier, because in half of the sessions of both training and test sets, the affective value of a proximal CS fractal will be positive, while it will be neutral in the remaining half. If Pavlovian conditioning in humans does not invoke MB computations, such a classifier would not be able to successfully decode the identity of the proximal stimulus.

In support of MB value computations during Pavlovian conditioning, we found evidence for encoding of stimulus–stimulus associations in two regions of interest, in the OFC and the dorsal striatum. These brain areas were found to contain information suggesting predictive representations of the identity of a subsequent stimulus with which that cue had been associated.

## Results

### Behavioral results

At the beginning of each block, participants rated the subjective pleasantness of each of the liquid USs. We found that participants rated the juice more favorably than the neutral liquid. This was expected, given that participants were able to select their favorite juice (from a panel of 2 juices) as the reward to be used in the study. They rated the juice positively at the beginning of the study (intercept for juice, *t*_*lme*_(74) = 6.46, *p* = 1*e* − 10), but liked it less towards the end of the study (main effect of session, *t*_*lme*_(74) = −3.73, *p* = 4*e* − 04). Nevertheless, they still rated the juice positively before the beginning of the last block (intercept for juice ratings, *t*_*lme*_(25) = 3.11, *p* = 0.005). Participants rated the water as affectively neutral at the beginning of the study (intercept of water, *t*_*lme*_(74) = 1.15, *p* = 0.26), and rated it less favorably towards the end of the study (main effect of session, *t*_*lme*_(74) = −2.26, *p* = 0.03). Nevertheless, they still rated it as affectively neutral at the beginning of the last block (intercept for water, *t*_*lme*_(25) = −.13, *p* = .9).

### Evaluative ratings of the CS fractals

**Fig. 2:**
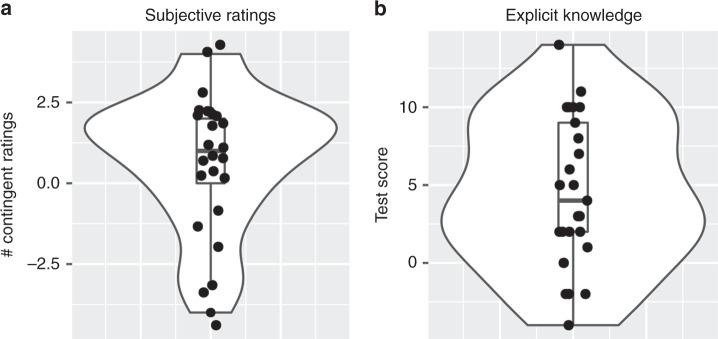
Behavioral measures of learning. **a** Consistent with model-free Pavlovian learning, participants’ subjective value ratings of CS fractals changed in concordance with Pavlovian contingencies, relative to their baseline ratings before Pavlovian conditioning. Plotted are the total number of CS fractal rating changes (after—before) contingent with Pavlovian associations. **b** Consistent with model-based Pavlovian learning mechanisms cognitive maps of Pavlovian contingencies, participants performed above chance on a test for explicit knowledge of Pavlovian associations. See Supplementary Fig. 1 for descriptive plots of ratings and test-score, grouped by stimulus type. Violin plots show mirrored density plot of behavioral results, boxplots show; Tukey-style box and whisker plots show the median, two hinges and two whiskers of data; Dots show individual participant results

As a measure of acquired subjective value of the CSd fractals, participants provided a liking rating for the CSd fractals at the end of the experiment, and tested whether ratings of CSd fractals had changed from the baseline rating at the beginning of the study. As dependent measure, we counted for how many CS fractals the change in subjective ratings was consistent with Pavlovian contingencies (Fig. 2a and Supplementary Fig. 2). We found that on average participants’ ratings changed in concordance with the Pavlovian contingencies. That is, in comparison to baseline ratings of CSd fractals, fractals associated with juice (CSd+) received more favorable ratings, while fractals associated with water (CSd−) received less favorable ratings (main effect of CS-type, *t*_*lme*_(174) = 1.84, *p* = 0.034 (*t*-test, one-tailed), *M*_*CSd*+_ = .39 (*SE* = .18), *M*_*CSd*−_ = −.02(*SE* = .17)).

### Explicit knowledge of Pavlovian contingencies

To test for behavioral evidence for the encoding of explicit knowledge about the Pavlovian contingencies, we tested participants’ explicit knowledge of Pavlovian stimulus—outcome (CSp/d—US) and stimulus—stimulus (CSd—CSp) associations at the end of the experiment (Fig. 2b). Specifically, participants were asked to make a binary choice regarding which US or CSp, respectively, they would expect to follow each CS fractal. We found that participants performed above chance (50%) on the test (*t*(24) = 4.74, *p* = 8.1*e* − 05, *t*-test). Participants performed above chance on CSd—US associations (*t*(24) = 4.88, *p* = 5.6*e* − 05, *t*-test), as well as CSd—CSp associations (*t*(24) = 2.20, *p* = 0.037, *t*-test). Participants’ test scores of their explicit knowledge of Pavlovian contingencies did not correlated significantly with their change in subjective ratings of CSd fractals (*r* = 0.33, *p* = 0.11, Pearson).

### Pupil responses to conditioned stimuli

As in our previous Pavlovian conditioning study^19^, we also collected pupil responses from all participants (see Supplementary Fig. 6). Unlike in this previous study, we did not use these data to calculate an estimate of how quickly participants acquired Pavlovian associations, because this was outside the focus of the present study. Nevertheless, in support of participants successfully acquiring the Pavlovian association of the distal CS fractal, we found that pupil dilation was increased for CS+ fractals in comparison to the CS− fractals (linear mixed-effects model with fixed factor *stimulus* (CS+ vs. CS−) and random effect *subject* revealed a main effect for the fixed factor (*t*_*lme*_(1904) = −3.07, *p* = 0.002).

### fMRI results

In our analyses of fMRI data, we focused on two brain regions, the striatum and the OFC, because of the established contributions of these areas in Pavlovian conditioning and reinforcement learning.

### ROI analysis of the striatum

The striatum is of interest, because of the established contribution of the ventral striatum to appetitive Pavlovian learning^20,21^, where it has been found to encode the value of Pavlovian conditioned stimuli^22–24^. The dorsal striatum plays a well-characterized role in reinforcement-learning^25,26^, with a double dissociation between medial and lateral dorsal regions in both rodents^27–29^ and anterior medial and posterior lateral regions in humans^30^ for goal-directed and habitual behavior, respectively, or, somewhat synonymously, with MB and MF reinforcement-learning^31–33^.

Whether a similar double dissociation within the human striatum exists for MB and MF Pavlovian learning is an open question. Given the established encoding of expected Pavlovian value signals in the ventral striatum, we predicted we would find such signals for Pavlovian CSs in our paradigm. Moreover, we hypothesized that we would find within the striatum evidence for the encoding of a cognitive map. One possibility is that such a map for Pavlovian conditioning would be represented in the ventral striatum alongside value signals encoded there. Alternatively, the cognitive map could be encoded in the caudate nucleus, on account of existing evidence supporting its involvement in goal-directed processes and MB encoding during instrumental conditioning^34,35^, as well as in human executive functions more generally^36,37^.

**Fig. 3:**
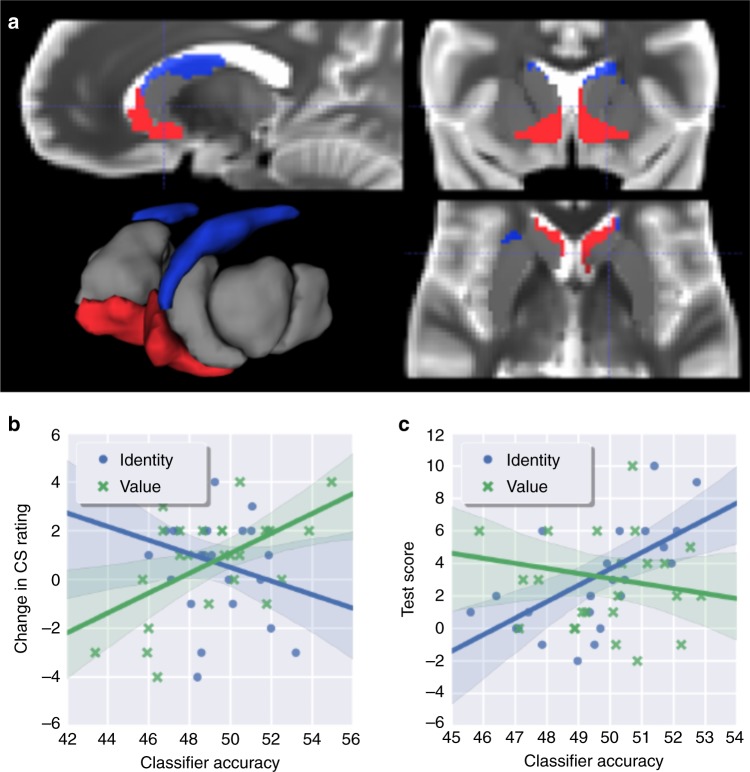
Results of ROI analyses of striatal representations. **a** Definition of functional striatal zones according to a previous meta-analysis^37^. The nucleus accumbens is highlighted in red, the body of the caudate is highlighted in blue. **b** Stimulus value classifier accuracy in nucleus accumbens correlates with changes in participants’ ratings of CS fractals (*r* = 0.58, *p* = 0.003, Pearson). **c** Stimulus identity accuracy in the body of the caudate nucleus correlated with participants’ explicit knowledge of CS-US associations (*r* = 0.64, *p* = 6*e*−4, Pearson). Violin plots show mirrored density plot of behavioral results, boxplots show; Tukey-style box and whisker plots show the median, two hinges and two whiskers of data; Dots show individual participant results

We determined that the spherical searchlight procedure commonly used for cortical analyses (including our analysis of OFC fMRI results here) is not as appropriate for sub-cortical structures such as the striatum, because a number of neuranatomically and functionally distinct regions (including ventricles) are tightly packed together in the basal ganglia, and the shape of these structures is highly irregular and non-spherical. Thus, if using a standard spherical searchlight procedure in the striatum, there is a substantial risk of the searchlight decoding from voxels positioned across anatomical boundaries, for which the interpretation would be difficult. That said, a searchlight is arguably more appropriate for cortical regions, where the risk of decoding across functional neuroanatomical boundaries is less severe (albeit still possible). In our ROI analysis of the striatum, we relied on an a-priori functional parcellation of the striatum into five functional zones^37^ (see Fig. 3a).

### Decoding value predictions from the striatum

We first tested for encoding of predictive value signals in the striatum. We trained a classifier to decode the value of the CSp based on activity patterns present at the time of presentation of the CSp. We then attempted to decode the value of the CSp based on the activity patterns present at the time of presentation of the CSd. This was done separately for each striatal ROI. We could not significantly decode value signals from any of the 5 striatal ROIs at *p* < 0.005, including the ventral striatum. Next, we tested for the extent to which the decoding accuracy of stimulus value signals across the striatal ROIs was correlated with the degree to which participants showed evidence of evaluative Pavlovian conditioning as indexed by the degree of change in the liking ratings for the distal CS cues from pre-learning to post-learning. We found that participants’ change in fractal ratings was significantly correlated with decoding accuracy of stimulus value in the ventral striatum (Fig. 3b, *r* = 0.58, *p* = 0.003 (Pearson); Bonferroni corrected across striatal ROIs at *p* = 0.013), but not in other striatal regions. This suggests that the degree to which the ventral striatum reliably encodes expected value depends on the extent to which participants manifest behavioral evidence of value-learning.

### Decoding identity predictions from the striatum

We then tested for encoding of stimulus–stimulus expectancies in the striatum. The analysis approach was identical to that used to test for encoding of predictive value signals in the striatum, except that the classifier was trained and tested on the identity of the CS fractals, rather than their values. Across the five striatal ROIs, we again did not find significant encoding of the identity of the CSp at the time of presentation of the CSd at *p* < 0.005. Next, we tested for correlations between the decoding accuracy of the identity of the CSp and explicit knowledge of Pavlovian contingencies across participants. We found a significant correlation between explicit knowledge of the Pavlovian contingencies and the accuracy of the stimulus identity classifier in the body of the caudate (Fig. 3c, *r* = 0.64, *p* = 6*e* − 4 (Pearson); Bonferroni corrected at *p* = 0.003), but not in the ventral striatum ROI (*r* = −0.23, *p* = 0.263, Pearson). Post-hoc tests revealed that the correlation between identity classifier accuracy in the body of the caudate and test scores for both CSd-US (*r* = .55, *p* = 0.004, Pearson) and CSp-US (*r* = .56, *p* = 0.003, Pearson) knowledge. At the same time, we did not find a correlation between knowledge of CSd-CSp assocations and identity classifier accuracy.

### Ventral striatum selectively predicts stimulus value

We explored whether the correlation between participants’ liking ratings and decoding accuracy of the value classifier accuracy was significantly greater in the ventral striatum compared to the body of the caudate. To statistically test for such a selectivity, we used a linear mixed-effects model, predicting classifier accuracy based on the fixed effects striatal *region* (ventral striatum vs. body of caudate) and participants’ liking *ratings*. In support of a selective involvement for the ventral striatum in containing information about expected value, this analysis yielded a significant interaction effect of region by rating (*t*_*lme*_(23) = −2.45, *p* = 0.023).

### Dorsal striatum selectively predicts stimulus identity

We explored whether the correlation between participants’ explicit knowledge of Pavlovian contingencies and the decoding accuracy of the identity classifier accuracy was significantly stronger in the body of the caudate than the ventral striatum. To statistically test for such a selectivity, we used a linear mixed-effects model, predicting classifier accuracy based on the fixed effects striatal *region* (ventral striatum vs. body of caudate) and participants’ *test scores*. In support of a selective involvement for the body of the caudate in containing predictions about subsequent stimulus identity: The statistical test yielded a significant interaction effect of region by test score (*t*_*lme*_(23) = 3.33, *p* = 0.003).

### Spherical searchlight analysis in the OFC

The OFC has also been implicated in appetitive Pavlovian conditioning in non-human animals^38–40^. Predictive reward signals have been reported in human OFC during Pavlovian conditioning^8,41^. Lending support to an involvement of MB mechanisms in Pavlovian learning, some of these signals have been found to be devaluation sensitive^41,42^. Beyond value signals, there is evidence for the encoding of other types of information not related to reward in the central OFC, including the identity of a US, at the time of presentation of an associated CS^17,18^ and the categorical identity of potential goal objects^43^. These findings support the possibility that the OFC may represent characteristics of anticipated stimuli beyond their rewarding properties, such as identity, which would allow it to play a role in encoding a map of Pavlovian contingencies, consistent with a prominent theoretical proposition implicating OFC in encoding a flexible cognitive map^44^.

Yet, sensory features in those prior studies relate specifically to associations formed with an affectively significant US. In order to establish whether the OFC is involved in encoding a more general and flexible cognitive map as opposed to exclusively mediating the learning of associations between arbitrary stimuli and affectively significant stimuli, it is necessary to demonstrate that the OFC encodes relationships between arbitrary stimuli that are not potent reinforcers in their own right. In the present study we aimed to test whether OFC encodes the identity of stimuli which have been arbitrarily associated with other stimuli without preexisting affective significance, which would be consistent with the encoding of a flexible cognitive map of stimulus–stimulus associations.

### Decoding identity predictions from the OFC

**Fig. 4:**
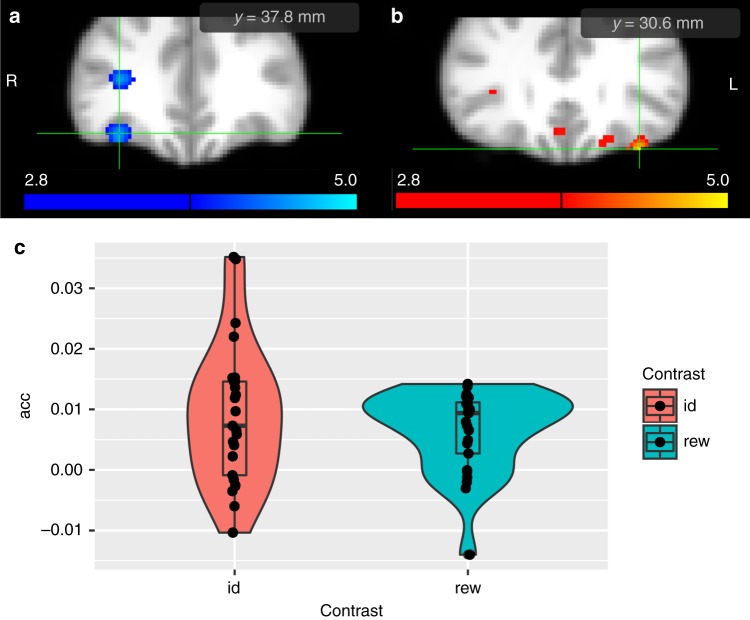
Searchlight results for orbitofrontal cortex. **a** When trained on CSp fractals and tested on CSd fractals, the stimulus identity (id) classifier accuracy was above chance in right central frontal orbital cortex (*xyz* = [34.2,37.8, −16.2]). **b** When trained and tested on CSd fractals, the stimulus value (rew) classifier accuracy was above chance in the lateral orbitofrontal cortex (*xyz* = [−34.2,30.6, −23.4]). For display purposes, we applied a threshold of *p* < 0.005. **c** Classifier accuracies for reward and identity classifiers in individual participants

We also predicted that we would find evidence for the involvement of the OFC in containing information about the identity of subsequent stimuli in the associative chain. Using a whole-brain searchlight procedure but restricting our searchlight results to an anatomical ROI of the bilateral OFC, we trained the classifier on patterns of activity elicited by the presentation of the CSp fractal, to then test its performance in decoding the identity of the CSp, based on the BOLD response to the presentation of the CSd fractal. As predicted, we found that the stimulus identity classifier showed above-chance accuracy in central the OFC (cOFC; *xyz* = (34.2,37.8, −16.2), *t* = 3.99(*p* = 2.7*e*−04, *t* − *Test*), *p*_*SVFDR* _< 0.05, *p*_*tfce* _< 0.05), indicating that the cOFC contained information about the expected identity of the CSp fractal at the time of presentation of the CSd fractal, consistent with a role for this region in representing stimulus–stimulus associations (Fig. 4a; classifier accuracies for each individual participant are shown in Fig. 4c; a similar plot showing the accuracy of individual cross-validation folds per subject is shown in Supplementary Fig. 7). We further tested for a correlation across participants between identity classifier accuracy in this cOFC cluster, and behavioral measures of explicit knowledge of Pavlovian associations. No significant decoding accuracy-behavior correlations were found (*r* = 0.24, *p* = 0.238, Pearson), nor was decoding accuracy correlated with changes in subjective ratings of CSd fractals (*r* = −0.13, *p* = 0.534, Pearson).

### Decoding value predictions from the OFC

We predicted a role for OFC in encoding the expected value of conditioned stimuli. As above, we used a whole-brain searchlight, but restricted our results to an anatomical ROI of bilateral OFC. We trained and tested the classifier on patterns of activity elicited by presentation of the CSd fractal, because it was the earliest predictor of reward delivery, hence relating our results to previous studies with canonical Pavlovian conditioning paradigms. As predicted, we found that the stimulus value classifier showed above-chance accuracy in OFC (*xyz* = (−34.2, 30.6, −23.4), *t* = 4.7(*p* = 4.5*e* − 05, *t* − *Test*), *p*_*SVFDR*_ < 0.05, *p*_*tfce* _< 0.05), consistent with a role for this region in representing value predictions (Fig. 4b). Classifier accuracies are shown for each individual participant in Fig. 4c. We further tested for a correlation across participants between value classifier accuracy in the lOFC cluster, and behavioral measures of subjective value ratings of Pavlovian CS fractals. We found no significant correlation between decoding accuracy with subjective ratings (*r* = −0.2, *p* = 0.327, Pearson), nor was decoding accuracy correlated with participants’ explicit knowledge of contingencies (*r* = 0.09, *p* = 0.673, Pearson).

### Classifier performance outside OFC and striatum

For completeness, effects found outside these specific ROIs at uncorrected thresholds are described in Supplementary Tables 1–6. These tables also provide results for alternative training/testing schedules of classifiers across proximal and distal CS fractals.

## Discussion

The aim of the present study was to determine if the brain encodes a cognitive map of stimulus–stimulus associations during Pavlovian conditioning. For this we utilized a sequential conditioning paradigm in combination with multivoxel pattern analysis of fMRI data. We found evidence for encoding of stimulus–stimulus associations in two regions of interest: the striatum and the orbitofrontal cortex. These brain regions were found to contain information suggesting predictive representations of the identity of a subsequent stimulus with which that cue had been associated.

Utilizing a region of interest analysis, we found contrasting roles for two distinct regions of the striatum: the body of the caudate was implicated in encoding stimulus–stimulus associations, conditional on the degree to which a participant could report explicit knowledge of Pavlovian associations. On the other hand, value signals were detectable in the ventral striatum, conditional on the degree to which participants manifested evidence of evaluative conditioning to the conditioned stimuli. These findings support a role for distinct regions of the striatum in encoding different aspects of stimulus characteristics during Pavlovian conditioning. It is important to note that our results do not allow us to draw conclusions as to whether ventral striatal value signals are the result of model-based or model-free learning, as either or both mechanism could be responsible for the acquisition and expression of value signals in that region.

A post-hoc analysis further revealed that stimulus classifier accuracy in the dorsal striatum was specifically correlated with stimulus-outcome knowledge, suggesting that the quality of model-based activity patterns in this region are associated with the ability of participants to remember stimulus-outcome associations explicitly after the end of the final session. This finding is important because a model-based Pavlovian agent ultimately needs to compute the value of different states of the world. To accomplish this it would be necessary to integrate knowledge of successive state-space transitions with knowledge about where in the state-space a rewarding outcome will be delivered. Here we found that the more discriminable representations of stimulus–stimulus associations were in the caudate nucleus, the better participants’ explicit knowledge of the identity of the outcomes linked to those stimuli. This suggests that participants can actively utilize stimulus–stimulus knowledge in the dorsal striatum in order to determine states of the world ultimately leading to valuable as opposed to less valuable outcomes.

Our results contrast with prominent theories^4^ and algorithmic approximations^5^ of Pavlovian conditioning that utilize model-free mechanisms (cf ref. ^10^). MF learning does not require knowledge of stimulus–stimulus associations to implement conditioning, but rather depends exclusively on the assignment of a scalar value signal to the CS. Instead, our results suggest that the cOFC and the body of the caudate nucleus contribute to representing a cognitive map of Pavlovian contingencies. By contrast, the lOFC and nucleus accumbens contribute to encoding CS value, which could depend on either MB or MF learning. Our results suggest that the distinction between MB and MF RL might usefully apply in Pavlovian conditioning above and beyond previous findings that have reported similar mechanisms in instrumental conditioning.

Our findings about distinct representations in the striatum are of interest in relation to a previously posited division of labor between ventral and dorsal striatum in instrumental conditioning: the actor-critic architecture^45^. According to this hypothesis, the ventral striatum acts as a critic that provides feedback (via a prediction error signal) to a dorsal striatal actor about whether chosen actions were appropriate^46^. Our results show that a similar division of labor is also present during Pavlovian conditioning, where, by definition, there is no actor. That is, this suggest that the dorsal striatum is involved in predicting future states, even without an opportunity to influence state transitions.

It is possible that our dorsal striatal finding resulted from participants’ belief in agency, i.e., that they have an opportunity to act in order to affect which outcome they will receive. This would be consistent with the existing proposal of a division of labor between a ventral critic and a dorsal actor^46^. However, while there exists a possibility that participants operated under the erroneous assumption that reward delivery was contingent upon the production of instrumental responses (such as fixating their gaze upon the CS fractal), our task instructions did not promote this false belief. Furthermore, because reward delivery was not contingent on instrumental behaviors, it is unlikely that reward delivery would have systematically reinforced any specific behavior.

Our interpretation of the striatal findings rests on the assumption that it is meaningful to interpret below-chance classifier accuracies in our study. That is, in participants with inconsistent changes in ratings of CS fractals or little knowledge of Pavlovian contingencies, the accuracy of the stimulus value in the nucleus accumbens and stimulus identity classifier in the caudate nucleus, respectively, were reliably below chance. MVPA is considered an information-based analysis technique^47^. Thus, testing single-subject accuracy can theoretically never be below chance level, because it measures the amount of information present^48,49^. However, the finding of decoding accuracies at below chance level in the striatum can be very plausibly explained in relation to our present findings. Specifically, these results emerge naturally from the combination of a cross-decoding procedure with leave-two-sessions-out cross-validation^48,50^. In our ‘cross decoding’ procedure we trained on one set of conditions (CSp in one half of learning sessions), and tested on another set of conditions (CSd in other half of learning sessions). Below chance accuracy could occur as a result of this cross-training procedure under the situation where the participants have incorrectly learned the associations between cues and rewards and/or cues and their explicit knowledge of the associations with subsequent cues. If in a given session, a participant has learned incorrect associations, and these are subsequently corrected on subsequent sessions, then the classifier will incorrectly classify the learned assignments from session to session. We tested this hypothesis by running simulations (see section Simulations of Supplementary Information and Supplementary Fig. 5), which confirmed that below-chance accuracy would be expected, if participants form the incorrect outcome anticipation in a majority of trials in some of the learning sessions.

We also implicated the OFC in encoding predictive information about stimulus identity. These findings build on previous fMRI results about stimulus identity coding^17,18,43^, as well as findings in rodents implicating the OFC in model-based inference^51^. Howard et al.^17^ reported unconditioned stimulus identity is present in the OFC. Further evidence implicates identity-based error signals in the midbrain in the formation of these identity representations^52^. However, those prior results leave open the extent to which the OFC encodes flexible non-reward associations during Pavlovian conditioning. This is because an unconditioned stimulus might have privileged access to the OFC, given its role in signaling rewarding or punishing consequences. Here, we found that associations involving even arbitrary stimuli with no prior value, can be encoded in the OFC during sequential Pavlovian conditioning. Our findings are, together with that of Howard et al., consistent with the possibility that the OFC encodes a flexible cognitive map of Pavlovian stimulus–stimulus contingencies. These findings are consistent with a role for the OFC in encoding a flexible cognitive map^44^.

The OFC also contained information about upcoming reward. Specifically in a region of lateral OFC, we could decode whether or not the distal cue was associated with the subsequent delivery of juice vs. the neutral non-rewarding liquid. These findings are consistent with an extensive literature in both humans and other animals implicating the OFC in encoding value predictions^8,17,38–41^.

While we found that the stimulus identity classifier performance was above chance in the OFC, we did not find a correlation between this classifier’s accuracy and participants’ explicit knowledge of Pavlovian contingencies. This stands in contrast to our finding that classifier accuracy in two striatal regions distinctly correlated with two behavioral measures of learning during Pavlovian conditioning. While we did not predict an absence of a correlation between classifier accuracy in OFC and behavioral measures, it is nevertheless interesting to speculate how this finding relates to previous findings^53,54^. One possible explanation is that while the OFC is involved in encoding the relationship between stimuli and expected reward, as well as in encoding stimulus–stimulus associations, these representations are not directly utilized to drive behavior in the OFC. Instead, perhaps these signals are passed to other structures which parse the information to drive behavior, the striatum being a prime candidate, given that striatal signals were found to be directly correlated with behavior. Along these lines, an important direction for future work would be to attempt to characterize the relative contributions of these areas as a function of time, both within trials and across trials as a function of Pavlovian learning.

An important caveat about our OFC results is that classifier performance though statistically significant, was relatively low. The high resolution protocol we utilized (1.8 mm voxels isotropic) may have contributed to decreased classifier performance as signal to noise is sacrificed to gain higher spatial resolution^55^. Yet, low OFC decoding accuracy is not unique to the present study, as it has been found more widely across prefrontal cortex even in conventional scanning protocols^56^. Nevertheless, because of this limitation, additional replications of these effects will be needed to assess their robustness.

A possible confound in this study is that when the classifier was trained on BOLD responses to the proximal stimulus and tested on the distal stimulus, decoding success could be influenced by a leaking prelonged BOLD response from the proximal stimulus to the distal stimulus, rather than by a statistically independent response to the distal stimulus. We attempted to rule out this possibility by ensuring that the experimentally induced jitter between the two stimulus events effectively decorrelated their associated hemodynamic responses. Indeed we confirmed that the correlation between modeled canonical hemodynamic responses at these two time points was very low. However, we cannot completely exclude the possibility that subtle temporal auto-correlations could contribute to the results.

To conclude, stimulus–stimulus predictions were found during Pavlovian conditioning in humans in both the striatum and orbitofrontal cortex. We further found that while the ventral striatum encoded anticipatory value representations, the dorsal striatum encoded anticipatory identity representations. This finding sheds new light on the division of labor between the two striatal areas and further suggest that neural representations during Pavlovian conditioning are much richer than hitherto assumed. Rather than exclusively depending on model-free computations, it appears the brain may also utilize a richer encoding of a cognitive map of a state space, even during Pavlovian learning.

## Methods

### Participants

Twenty-six (14 female) healthy volunteers participated in this fMRI study. The sample size was chosen based on a previous study with a similar paradigm^19^. Participants were free of neurological or psychiatric disorders and had normal or corrected-to-normal vision. Written informed consent was obtained from all subjects, according to a protocol approved by the Human Subjects Protection committee of the California Institute of Technology (Pasadena, CA). One participant had to be excluded from analysis because of a hardware failure during data acquisition. In the remaining sample, mean age was 25.76 years (minimum: 18, maximum: 33).

### Behavioral task

Human volunteers participated in a sequential Pavlovian conditioning paradigm^19^, in which they learned to associate two sequentially presented conditioned stimuli (fractal images), with either a pleasant (juice) or an affectively neutral (artificial saliva made of 25 mM KCl and 2.5 mM NaHCO3) flavor liquid (referred to as water). Details of the trial structure are shown in Fig. 1, and in ref. ^19^. The present study was inspired by the experimental design used in a previous study^19^, but the experimental design used in the present study was optimized for the aims of the present study: (1) the previous study included aversive trial outcomes (unconditioned stimuli), which were not the focus of the present study, (2) we reduced the number of incongruent trials compared to the previous study (because we were interested in decoding neural representation of conditioned stimuli, rather than finding reward prediction errors in incongruent trials), and above all (3) the experimental contingencies were designed such that we could train/test machine learning classifiers on two orthogonal classification tasks: classification based on stimulus identity vs. based on stimulus value.

Each trial began with the presentation of a fixation cross on a dark gray screen. Then, the first fractal (CSd) image was presented in one of 8 random locations, followed by the presentation of another fractal image (CSp) in one of the remaining 7 locations, also chosen at random. The merits of varying cue locations has been discussed previously^19^. The second fractal image presentation co-terminated with the end of delivery of liquid. At the end of a trial, participants were asked to swallow the delivered liquid. The fixation cross remained on the screen throughout the trial, but disappeared during inter trial intervals. As in previous variations of this paradigm^19,57^, the deterministic proximal cue (CSp) was sometimes not delivered as predicted by the distal cue (CSd). Instead, oddball CS fractals were presented (CS-S vs. CS-T), inducing a valence reversal, and therefore both positive and negative prediction errors, capturing the core feature of the temporal difference algorithm: learning via prediction errors induced by sequential predictors. However, to optimize the existing paradigm for MVPA, the expectation evoked by the distal cue would be reversed by the proximal cue in only 20% of trials (24 trials total). Thus, in optimizing the present study for the purpose of MVPA analyses, the number of unexpected CSp fractal presentations was reduced. While the relatively smaller number of unexpected CSp fractals precluded the analysis of neural correlates of reward prediction errors, this probabilistic sequential trial structure was retained in oder to make the task more engaging for participants.

The experiment consisted of four sessions, lasting approximately 16 min each. Each session was composed of 30 trials, yielding a total of 120 trials. In all four sessions, the same two fractals were used as CSp fractals, referred to as CS-X and CS-Y. In contrast, two unique CSd fractals were chosen for each session (see Fig. 1). Critically, the valence of CSp fractals was reversed between consecutive sessions. Thus, in half of the sessions CS-X was associated with juice delivery, while it was associated with the delivery of neutral liquid in the other sessions. This counterbalancing of CSp valence across sessions allowed for the cross-validated training of two different classifiers. The first classifier was trained to classify based on stimulus identity (i.e., CS-X vs. CS-Y). The second classifier was trained to classify based on valence, irrespective of CSp identity (i.e., CS+ vs. CS−). Put another way, by alternating the CSp to juice and water associations across blocks, we ensure that areas identified as encoding CSp identity are doing so independently of the specific outcome with which that CSp is associated on a given block.

### Participant instructions

Before participants signed up for the experiment, they were informed that participation was conditional on their commitment to not consume any food for the last 4 h before the experiment. This was done to induce a subtle state of food deprivation, in order to increase the value of the pleasant juice liquid. At the same time, we aimed to avoid rendering participants thirsty, to ensure that the artificial saliva was perceived as affectively neutral relative to the juice. For this reason, participants were encouraged to drink water during the 4 h deprivation period to ensure that they remained hydrated.

Before participants entered the scanner, they received the following verbal task instructions: In each trial, an image will appear on the screen, followed by a second image, which will be followed by the delivery of a liquid. Each image will help you predict what kind of liquid will be delivered. There will be four sessions.

### Apparatus

The pleasant and neutral tasting liquids were delivered by means of two separate electronic syringe pumps. These pumps pushed 0.75 ml of liquid to the participants’ mouth via clear PVC plastic tubes (http://www.freelin-wade.com; outside diameter, 8 mm; inside diameter, 4.8 mm), the other ends of which were held between the participants’ lips like a straw, while they lay head first supine in the scanner.

### Subjective ratings

Before the experiment, and in between experimental sessions, participants were asked to indicate on a scale from −3 to +3 how they liked the fractal images, the degree of hunger, thirst, and how much they liked the reward (juice) and neutral outcome (results are shown in Supplementary Fig. 8). Throughout the experiment all rating scales were presented in form a horizontal bar on the screen, with equidistant tick marks in order to ensure the legitimacy of assuming an interval scale for the rating data (see ref. ^58^, for a discussion).

### Rating of unconditioned stimuli

At the beginning of the study, participants were asked to choose one of two juices (cranberry apple or apple juice, Trader Joe’s, Monrovia, CA) to be used as rewarding liquid throughout the experiment. This was done by having participants rate each juice, as well as the neutral outcome (artificial saliva, referred to as water) on a scale from −3 (strong dislike), to +3 (strong like).

### Rating of fractals

At the beginning of the experiment, participants were asked to rate all 22 fractals. This initial rating of fractals served two distinct purposes. First, the ratings were used as a baseline, against which to compare ratings of the same fractals after Pavlovian conditioning. Second, to ensure that only relatively neutral fractals were used as CS fractals during Pavlovian conditioning, the 10 fractals with the most neutral ratings were selected as CS fractals for Pavlovian conditioning. In order to avoid any systematic bias of stimulus ratings of different CS fractals (e.g., CS-X always the least favorite one in all participants), the 10 fractals were permuted before assigning them to different CS fractals. After each of the 4 sessions of Pavlovian conditioning, participants rated all of the 22 fractals again. We included the unused 12 other fractal images in these ratings, to make it harder for the participants to recall their initial baseline rating of each fractal.

In order to evaluate whether Pavlovian conditioning affected ratings of CS fractals, we calculated the difference between the rating of a fractal at the end of the experiment, minus the fractal’s baseline rating at the beginning of the experiment. To generate an overall measure of how Pavlovian conditioning affected fractal ratings, we calculated for each participant the sum total of changes in CS fractal ratings between the beginning and the end of the experiment. Rating changes of CS fractals associated with a neutral outcome were negated, so that this sum would represent a measure of contingency-dependent changes in CS fractal valence. We focused our analyses on fractals that acted as CSd fractals, because ratings of CSp fractals are difficult to interpret, due to their repeated valence reversal between consecutive sessions (see Fig. 1). In order to test statistically whether there was an overall effect of Pavlovian conditioning in the population, we applied a linear mixed-effects model, with the fixed factor CS-type (CS+ vs. CS−), and participant as a random factor.

### Test of explicit knowledge of stimulus contingencies

After the conclusion of the final learning session, we asked participants to engage in a test of their explicit knowledge of stimulus contingencies. Participants were not made aware of this test until they had completed all sessions of Pavlovian conditioning. The test presented an opportunity for participants to gain up to an additional $5. In each test trial, a probe CS fractal would first appear in the center of the bottom half of the screen, followed by the presentation of two target images next to each other in the upper half of the screen. In the case of a CSd fractal probe, the target images could either be two CSp fractals (test of stimulus–stimulus knowledge), or one image depicting a drop of juice, and the other a drop of water (stimulus-outcome knowledge). In the case of a CSp fractal probe, one image would depict a drop of juice, and the other a drop of water. That is, CSp fractals were not used as probes to test for explicit knowledge of stimulus—stimulus associations. Participants were asked to indicate which stimulus/outcome would follow the fractal image at the bottom of the screen, by selecting either the left or the right image at the top of the screen. After making a selection, participants were able to bet any amount between $0 and $1 (in increments of 25 cents) on their answer. This was done to ensure that they would answer earnestly. The test contained 12 questions regarding stimulus—outcome associations (8 CSd, 2 CSp, and 2 oddball CS fractals), and 8 question regarding stimulus—stimulus associations (8 CSd fractals). When tested on the CSp fractals, participants were instructed to answer based on the final Pavlovian conditioning session. The total test score was the sum of correct answers (i.e., excluding omissions and incorrect answers).

For statistical analysis of whether participants had explicit knowledge of either stimulus—outcome or stimulus—stimulus associations, we performed a *t*-test of whether the total test score of each participant was above 0.

### fMRI data acquisition

Functional imaging was performed on a 3 Tesla MRI system (Magnetom Tim Trio, Siemens Medical Solutions) located at the Caltech Brain Imaging Center (Pasadena, CA) with a 32-channel head receive array for all the MR scanning sessions. To reduce involuntary head motion, participants’ heads were securely positioned with foam pads.

Because the focus of our study was the orbitofrontal cortex (OFC) and striatum, we acquired T2*-weighted echo planar images (EPI), with coverage limited to the anatomical boundaries of the striatum and OFC while participants were performing the task (see Supplementary Fig. 9). Slices were positioned in order to cover both the ventral prefrontal cortex and the striatum. A total of 35 slices were acquired with a multi-band acceleration factor of 5, and an isotropic resolution of 1.8 mm. Other imaging parameters included: TR = 600 ms, TE = 30 ms, flip angle = 50 degrees, field of view = 180 mm, matrix = 100 × 100. Whole-brain high-resolution T1-weighted and T2-weighted structural scans (isotropic voxel size = 1.0 mm). Dual-echo gradient echo field maps were acquired to allow geometric correction of the EPI data. We discarded the first 3 EPI volumes before data processing and statistical analysis to allow for magnetization equilibration.

### fMRI data pre-processing

Pre-processing of functional MRI data was implemented in NiPype^59^, allowing the development of a pre-processing pipeline specifically tailored to the high-resolution fMRI data of subcortical structures. Functional images were corrected for participant motion, fieldmap corrected for geometric distortion, high-pass filtered (default FSL high-pass filter cutoff of 100 s), rigid-body co-registered to the participants T2-weighted structural image, and automatically denoised using independent components analysis and hierarchical fusion of classifiers (ICA-FIX)^60^. In order to achieve higher accuracy, the ICA-FIX classifier was trained on the present data set. Preprocessed functional images were diffeomorphically co-registered^61^ to the California Institute of Technology CIT168 brain template in MNI space^62^, using nearest-neighbor interpolation, leaving functional images in their native 1.8 mm isotropic resolution. No smoothing was applied to the data.

### Multivoxel pattern analysis

All multivoxel pattern analyses (MVPA) were performed in PyMVPA (version 2.5.0)^63^. For MVPA, we first fit a GLM with a separate regressor for each stimulus onset (three regressors per trials: (1) distal and (2) proximal CS fractals and (3) US onsets). Thus, instead of performing classification based on the volume closest to the presumed peak of the BOLD response, or averaging across a time-window centered around this peak, analyses were performed on the parameter estimates of the GLM^64^. We believe this approach is preferred, given the very high temporal resolution of our dataset (TR = 600 ms), and moderate amount of co-linearity achieved by the addition of temporal jitter to stimulus onset asynchrony (Supplementary Fig. 10). Before GLM estimation, we performed quadratic detrending of the fMRI time series^65^. GLM parameter estimates were normalized (z-score) before further classification analysis^66–68^. No orthogonalization was performed among the regressors.

The design of the experiment allowed two independent classifiers to be trained. One classifier was trained to classify CS fractals, either CSd or CSp fractals, according the identity of the CSp fractal in the current trial (CS-X vs. CS-Y). We refer to this classifier as the stimulus identity classifier. The second classifier was trained to classify CS fractals according to the valence of the CSp (CS+ vs. CS−), independent of CS identity (see “Task Description” above). We refer to this classifier as the stimulus value classifier. All classification analyses were performed with a linear support vector machine (SVM) classifier.

Classifier training and testing was done in a fully cross-validated manner with 4 folds. In each fold, a classifier was trained on the CS fractals of two of the four sessions, and its performance was tested on CS fractals from the remaining two held-out sessions. We chose this cross-validation approach to ensure that auto-correlations can be ruled as driving classifier performance. In fact, we had conducted a separate analysis to validate this concern: We found that if we chose to perform odd-even trial cross-validation, classifier performance was above chance, even in a permutation test. This confirms our suspicion that due to the temporal sequence of events in our experimental paradigm, cross-validation across learning sessions was necessary to guard against the effects of autocorrelations in the BOLD responses.

### Searchlight analyses

Whole brain searchlight analyses were performed with a spherical searchlight, with a radius of 3 voxels. The SVM cost/penalty parameter *C* was set to 1.0 for all searchlight analyses. The classification accuracy of each searchlight was assigned to the center voxel of the sphere. Before second-level analyses, individual accuracy maps were smoothed with a Gaussian smoothing kernel of 5.4 mm (FWHM; three times the voxel size). To test the global null hypothesis that there is no information in any subject in the test population^69^, a one-sample t-test was used to test whether classifier performance was above 50%, i.e., chance level^48^. We performed small volume FDR correction (SVFDR *p* < 0.05) of searchlight results. The OFC was defined according to the Harvard–Oxford anatomical atlas^70,71^. In order to avoid the potential limitations of cluster-based thresholding^72^, we also determined familywise error correction for results reported in the main Result section, by using small volume threshold-free cluster enhancement (tfce) and indicate whether an effect exceeded this threshold (*p*_*tfce*_ < 0.05).

### ROI analyses

In order to investigate the topography of anticipatory stimulus identity and reward representations in the human striatum, we performed a region of interest analysis. For this analysis within the striatum, we utilized an existing parcellation of the striatum into 5 non-overlapping functional zones^37^, based on differences in co-activation between striatal and non-striatal voxels across a wide range of psychological tasks and states^73^. For this analysis, we created a meta-classifier, implementing a pipeline consisting of a PCA-based feature dimensionality reduction, followed by classification by a support-vector machine (SVM).

We followed an unbiased algorithmic approach to the selection of the regularization parameter C in the SVM and the number of retained components after PCA-based dimensionality reduction. In doing so we went beyond the current practice in the fMRI literature to either not report these hyperparameters at all, or to select them according to one of several possible heuristics. Here, we selected 80% (*n* = 20) of participants for optimization of these hyperparameters, to then apply these hyperparameters during the analysis of the remaining 20% (*n* = 5) of participants. To ensure that performance of a classifier for a given participant was not affected by which subset of participants was included in this parameter optimization, we repeated this procedure for 1000 iterations. We found that this approach resulted in a modal value of 16 for the number of retained PCA components, and a modal value of 1 for the regularization parameter C in the SVM. While the main findings were stable from the first iteration, the non-significant correlations appear less stable until a higher number of iterations (Supplementary Figs. 11 and 12). We speculate that if an area does not represent information of use for a classifier, our optimization approach would result in a noisy hyperparameter selection, resulting in noisy estimates of classifier performance when these parameters are applied in the analysis of the remaining participants, and will eventually result in non-significant findings as well. Overall, had we not followed this iterative process, and instead had used e.g., standard k-fold cross-validation or had made heuristic choices for these hyperparameters, we may have erroneously reported false positive findings.

### Brain behavior correlations

Next, we tested whether classifier accuracy in any of the striatal ROIs correlated with either participants’ (1) change in ratings of CSd fractals (2) and test score for explicit knowledge of experimental contingencies. For this purpose we calculated the Pearson correlation between either behavioral measure and accuracies of either classifier. A correlation was deemed significant if it survived Bonferroni correction (p-value divided by the number of ROIs (five)).

We also tested whether there was a correlation between classifier accuracy in cortical areas and behavioral measures of Pavlovian conditioning. We limited this analysis to clusters that survived correction for multiple comparison (TFCE, see above). For this purpose, we defined clusters as all contiguous voxels that survived an uncorrected threshold of *p* < 005, and calculated the median classifier decoding accuracy in these voxels. As above, we then calculated the Pearson correlation coefficient between this median classifier accuracy within a cluster with either behavior measures.

### Code availability

Computer code used for preprocessing the data and analyzing the data is available in a publicly hosted software repository [https://github.com/wmpauli/mb_pavlovian_mvpa].

## Supplementary information

Supplementary Info

Peer Review

## Data Availability

Raw, de-identified MRI data are available at the Open Science Framework [10.17605/OSF.IO/CHFNW].
