# Supplementary material for: Evidence for model-based encoding of Pavlovian contingencies in the human brain
Source: Nat Commun. 2019 Mar 7;10:1099. doi: 10.1038/s41467-019-08922-7 (PMC6405831; doi:10.1038/s41467-019-08922-7)
Supplement: Supplementary file 1 — Supplementary Info [file 41467_2019_8922_MOESM1_ESM.pdf]

# Supplementary Information

## A Simulations

To demonstrate how below-chance classifier accuracy emerges from the combination of leave-out session cross-validation and within-subject variance in learning performance across sessions in our Pavlovian conditioning paradigm, we ran simulations on generated BOLD and behavioral data. Training and testing was done analogously to the above analyses of experimental fMRI data. For parsimony, a classifier was simply trained on a binary categorization task (e.g. corresponding to reward (CS+) and non-reward (CS-) trial types), with 4 simulated Pavlovian conditioning sessions, with 30 trials per session, 15 of each trial-type, as in the empirical study. In each iteration of the simulation, we generated a multivariate dataset with normal noise and specified means (with a mean of 0.0, and standard deviation of 1.0, using the normal_feature_dataset data_generator of the python mvpa2 library), yielding a dataset of 120 trails (samples), and 8 features (corresponding to the modal number of features in the above analyses of participant data).

To simulate within-subject variance in learning performance, we proceeded according to the following logic. In the experiment, we probed participants for whether they had acquired the Pavlovian contingencies in each of the sessions. For example, by asking them whether they would predict the delivery of juice or water after seeing a probe CS fractal. If participants answered this two-alternative question incorrectly, we assumed that they would also have made the incorrect prediction in a majority of trials from the relevant Pavlovian conditioning session. We refer to sessions for which participants answered incorrectly as "*poor*" learning sessions. In our simulations, we would therefore swap the classification target labels for a majority of the trials in a poor learn-

ing session. Because our volunteers participated in four sessions of Pavlovian conditioning, we further investigated the effect of any possible number (0 - 4) of poor learning sessions on classifier performance.

To illustrate the resulting impact on classifier performance (Supplementary Figure 1a), we simulated that participants may have entertained the incorrect prediction for various fractions $\psi^p$ of the total number of trials in poor learning sessions (.5 - 1.0, in .1 increments). In addition, we also accounted for the fact that even in *s*uccessful learning sessions, because participants could not know the Pavlovian contingencies in advance, they may still have entertained the incorrect prediction for a minor fraction of trails ($\psi^s$, .1, .2, or .3). In sum, simulated average cross-validated classifier performance for participant $n$ ($\hat{y}_n$) is a function ($F$) of this participant's learning performance measures for the four sessions ($X_{(n,1..4)}$), and fractions of trials with incorrect outcome predictions in successful and poor learning sessions ($\psi^s_n$ and $\psi^p_n$, respectively). To get a stable estimate of simulated classifier performance $\hat{y}$, we ran $N$ = 1000 simulated participants for a given set $X_{1..4}$ of learning performance measures (subscripts $n$ omitted to indicate that $X_{1..4}$ was held constant across $N$ iterations):

$$\hat{y} = \frac{1}{1000} \sum_{n}^{N} F(X_{1..4}, \psi^s, \psi^p). \tag{1}$$

This revealed that simulated classification accuracy is the worst (Supplementary Figure 1a), if participants completely fail to reverse in half of the sessions, because of the cross-validation with two leave-out sessions.

In a second step, we performed an exploratory regression analysis (Supplementary Figure 1b), attempting to predict the average cross-validated classifier performance in each participant

($y_n$), given each participant's learning performance across the four sessions ($X_{(n,1..4)}$). Specifically, we treated the fraction $\psi^p$ and $\psi^s$ of trials in which participants entertained the incorrect prediction during poor and normal learning sessions, respectively, as free parameters in a ordinary least sum of squares (OLS) optimization:

$$minarg_{(\psi^s,\psi^p)} SSR(X_{N,1..4}, \psi^s, \psi^p) = \sum_{n}^{N} (y_n - \hat{y}_n)^2 \qquad (2)$$

Note that the two free parameters ($\psi^s, \psi^p$) were constrained such that they were held constant across all $n$ participants and sessions, because we only had binary measures (e.g. correct vs. incorrect) for each participant and session, but no information regarding how close each participant was to acquiring the Pavlovian contingencies. This constraint severely limited the flexibility of the optimization procedure. Particle swarm optimization (PSO; pyswarm, version 0.6) with constraint support was used to find best-fitting parameters. To get an estimate for the robustness of the fit, we performed 100 iterations drawing random samples of 80% of participants in each iteration. On each iteration, particles were initialized with random starting values ($0 <= \psi^s < .5$, $.5 <= \psi^p 1.0$). For the stimulus identity classifier and participants' explicit knowledge of stimulus-outcome contingencies, this procedure yielded a mean $\psi^s$ = 0.38 (SD = 0.11), $\psi^p$ = 0.79 (SD = 0.13), $SSR$ = 2.76 (SD = 0.97) (Supplementary Figure 1b, right). For the stimulus value classifier and participants' change in CS fractal ratings, this procedure yielded a mean $\psi^s$ = 0.36 (SD = 0.09), $\psi^p$ = 0.84 (SD = 0.16), $SSR$ = 5.91 (SD = 2.09) (Supplementary Figure 1b, left).

## B Supplementary Figures

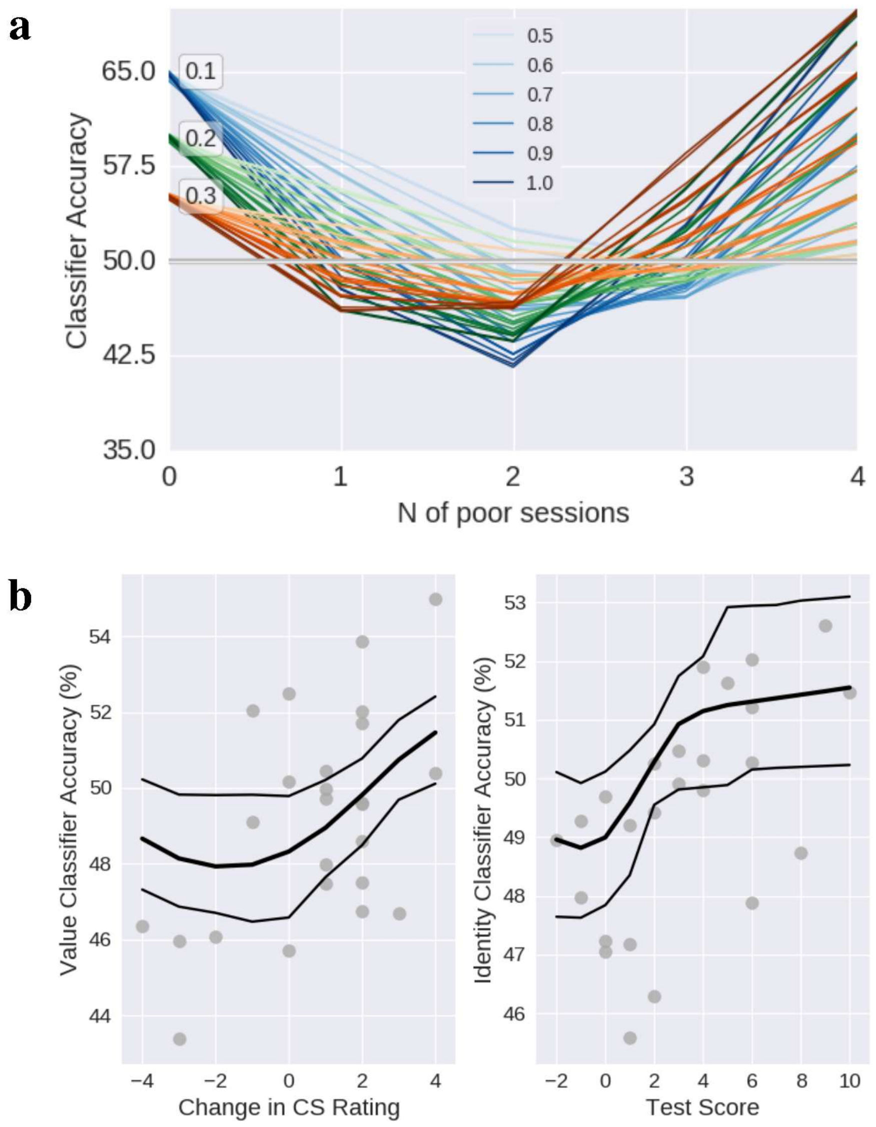


Supplementary Figure 1: Simulations demonstrate how below-chance classification accuracy occurs if participants are slow to adapt to Pavlovian contingency reversals. **(a)** Each line corresponds to a combination of learning performance (fraction of trials in which participants predicted the incorrect outcome) in successful ($\psi^s$, .1 - .3, different color-maps) and poor ($\psi^p$, .5 - 1.0, different shades within color-map) Pavlovian conditioning sessions. **(b)** Results of LSS fit of participants' data, for the value classifier and changes in CS ratings (left), and the identity classifier and test scores (right). Thick lines indicate the average across iterations, thin lines indicate 5th and 95th percentiles.

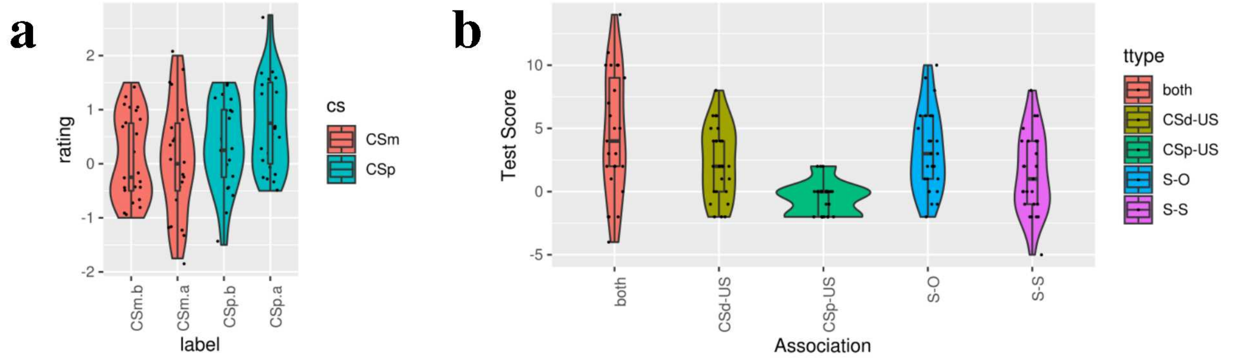


Supplementary Figure 2: **(a)** Individual pre- and post- session rating of CSd fractals on a seven point likert scala. CSm.b = $CS-_{before}$, CSm.a = $CS-_{after}$, CSp.b = $CS+_{before}$, CSp.a = $CS+_{after}$. **(b)** Individual test score totals and scores on subtests. Scores for S-S and S-0 subtests sum to 'both', CSd-US and CSp-US sum to S-0.

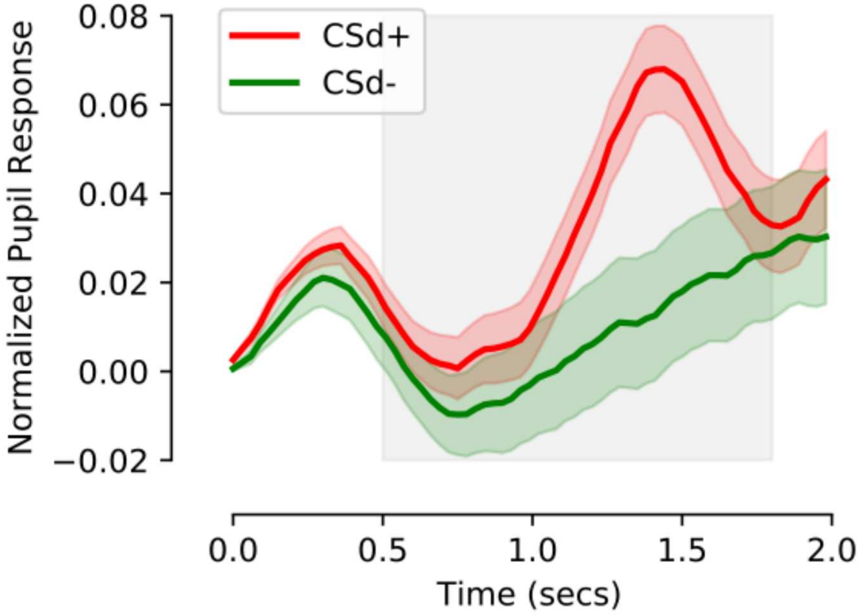


Supplementary Figure 3: We collected pupil responses from all participants. In support of participants successfully acquiring the Pavlovian association of the distal CS fractal, we found that pupil dilation was increased for CS+ fractals in comparison to the CS- fractals (linear mixed-effects model with fixed factor *stimulus* (CS+ vs. CS-) and random effect *subject* revealed a main effect for the fixed factor ($t(1904) = -3.07, p = 0.002$)

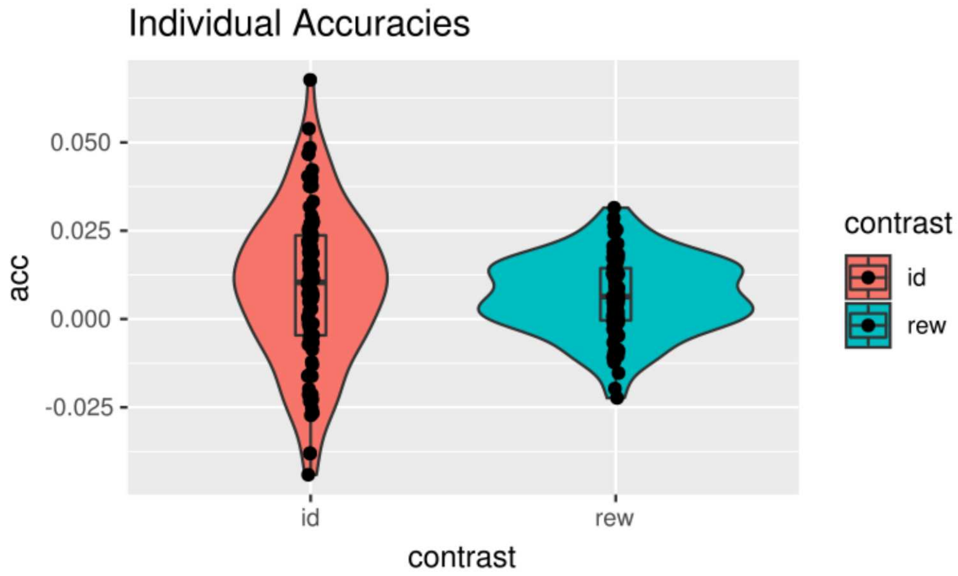


Supplementary Figure 4: Violin plots of classifier accuracies. This figure is similar to Figure 4 (c), except that classifier accuracy is plotted separately for each cross-validation fold (i.e. four dots per participant). These plots show that classifier accuracy varies across cross-validation folds.

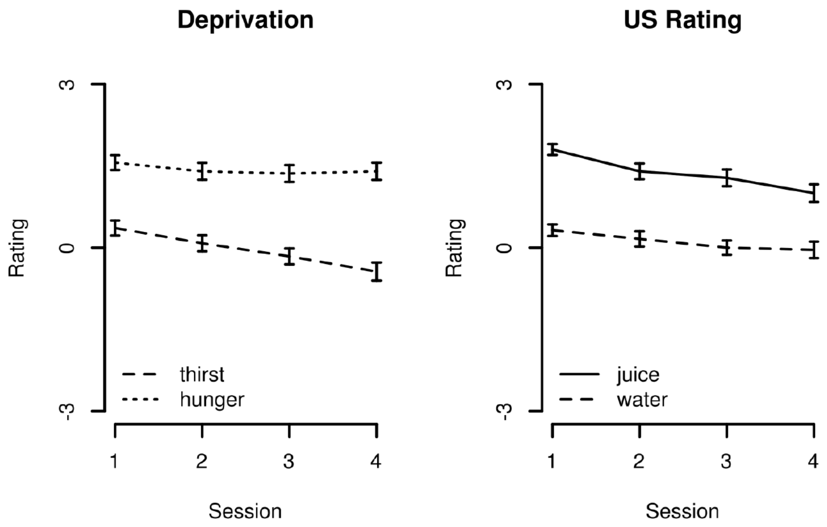


Supplementary Figure 5: Ratings of hunger and thirst (Left) and of US (juice and water; Right) on 7 point likert scale indicate that participants were hungry at the beginning of the study, and remained hungry throughout the experiment (significant intercept for hunger ($t_{lme}(74) = 5.1, p = 2.6e - 06$, but no main effect of session). Participants reported not to be thirsty at the beginning of the study, and reported to be even less thirsty at the end of the study (main effect of session, $t_{lme}(74) = -3.67, p = 5e - 04$), probably due to the delivery of a small amount of liquid at the end of each trial.

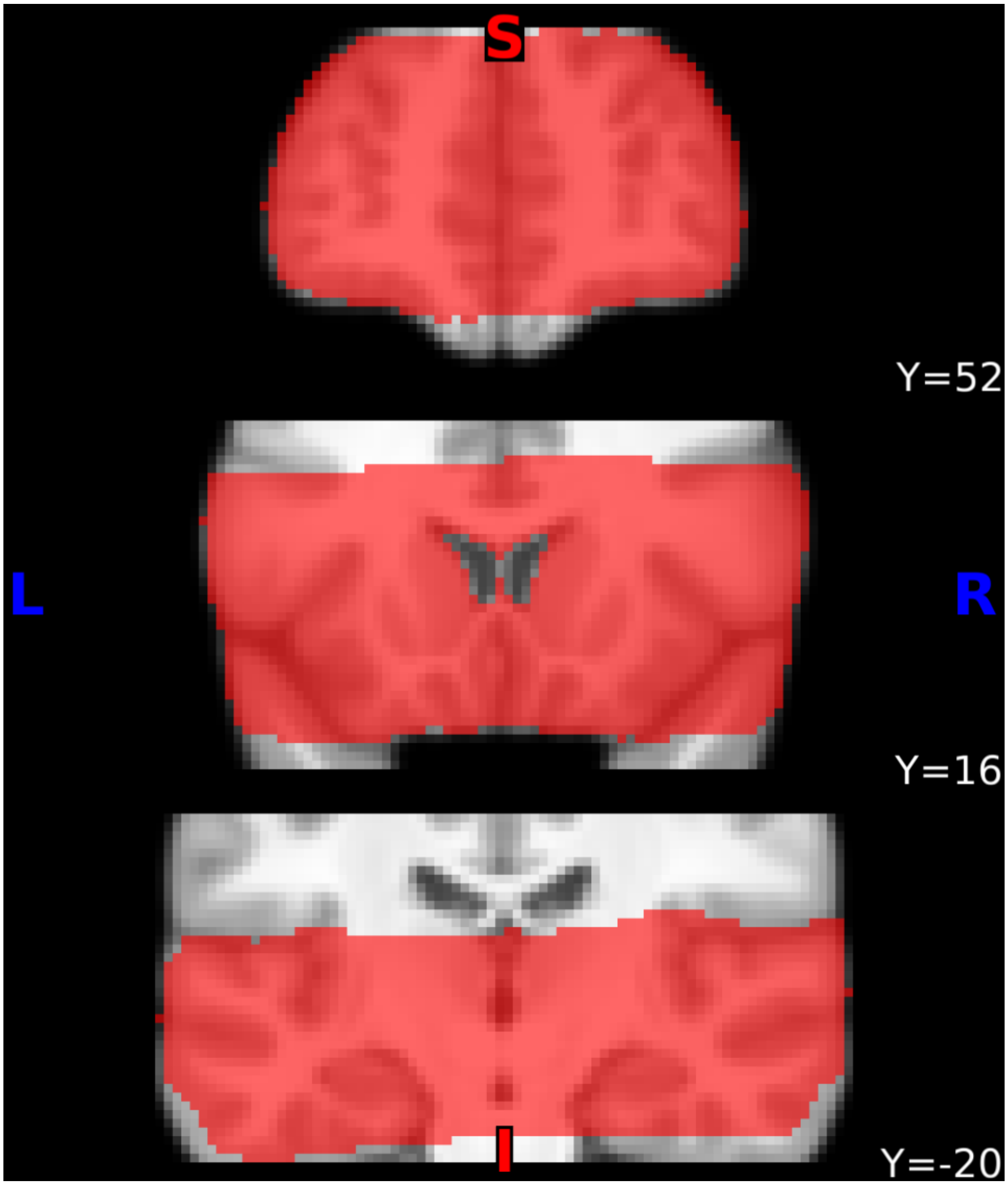


Supplementary Figure 6: Red overlay over three consecutive cortical slices of T1 image contrast indicates from which brain areas functional MRI data was acquired in all participants. Only voxels for which functional data was available for every participant were included in statistical analysis.

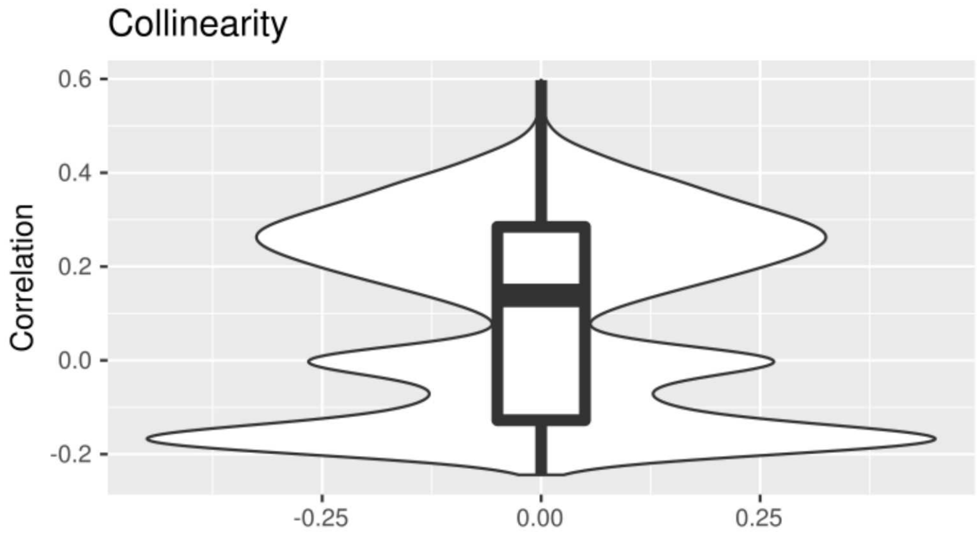


Supplementary Figure 7: Collinearity among regressors in the GLM used to create the data which classifiers were trained and tested on. The figure shows that collinearity among successive regressors was acceptable. In addition to training and testing classifiers across sessions, this further reduces the probability that above-chance classifier performance is merely an effect of residual activation (BOLD response) across trail events.

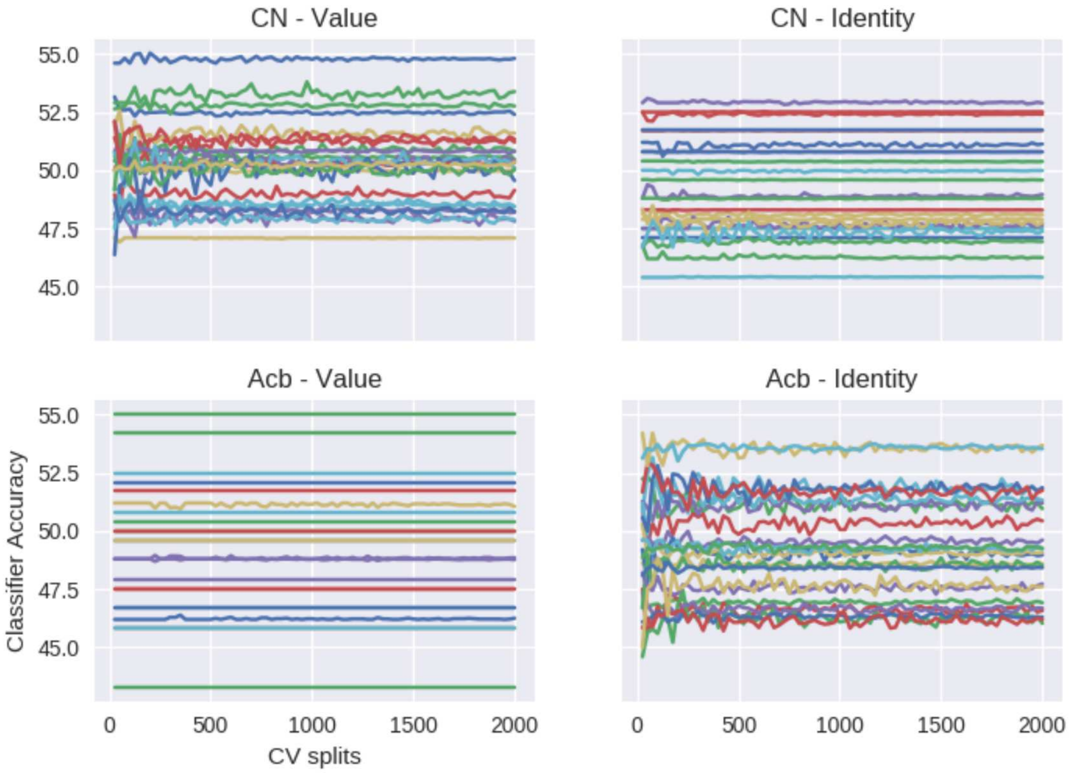


Supplementary Figure 8: Changes in classifier test accuracy across iterations of between-subject cross-validation for hyperparameter selection. While estimates for test accuracy of the value classifier in the nucleus accumbens (Acb) and the identity classifier in the caudate nucleus (CN) were relatively stable across iterations, this was less the case for the accuracies of the identity classifier in the Acb and the value classifier in the CN. This may indicate that our algorithmic approach to hyperparameter selection leads to relatively noisier parameter selection and classification results, if an area is not representing the information a classifier needs for classification.

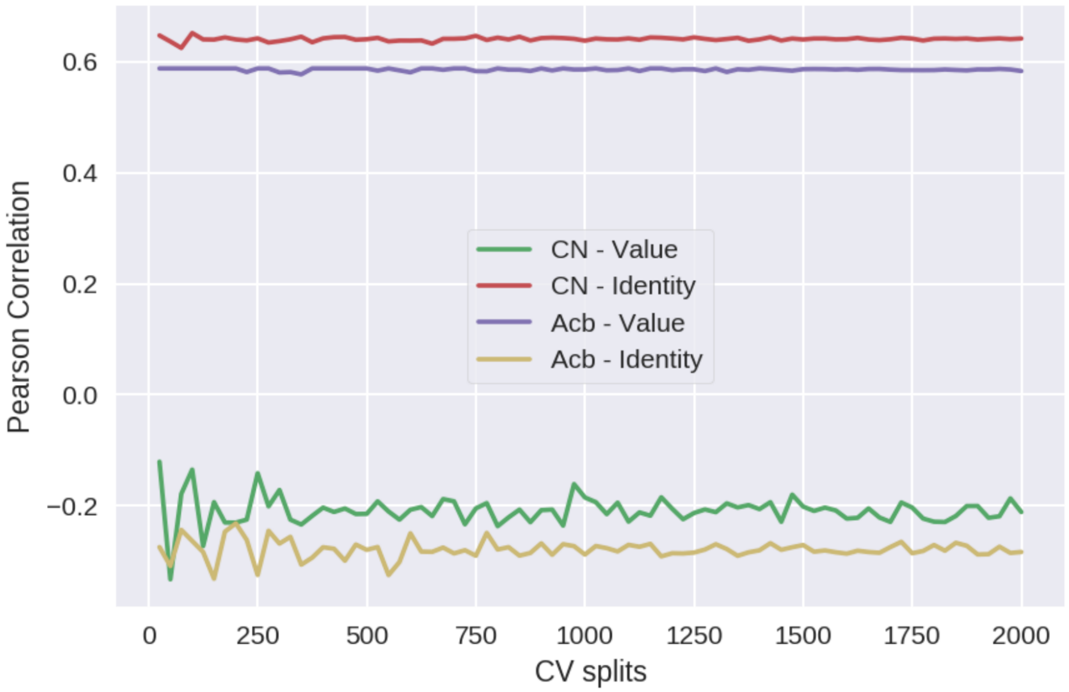


Supplementary Figure 9: Changes in correlations between classifier accuracy (see also Supplementary Figure 8 and behavioral performance across iterations of between-subject cross-validation for hyperparameter selection. While correlation estimates for the significant results reported in this manuscript (nucleus accumbens (ACb) value classifier accuracy and changes in subjective ratings; caudate nucleus (CN) identity classifier accuracy and explicit knowledge of Pavlovian contingencies) were relatively stable across iterations of cross-validation, this was less the case for the non-significant correlations (ACb identity classifier accuracy and changes in subjective ratings; CN value classifier accuracy and explicit knowledge of Pavlovian contingencies).

## C Supplementary Tables

| Region | Hemisphere | x | y | z | t | p |
|---|---|---|---|---|---|---|
| Temporal Pole | right | 34.2 | 9.0 | -19.8 | 3.95 | 3e-04 |
| | left | -59.4 | 10.8 | -7.2 | 3.06 | 3e-03 |
| Precentral Gyrus | left | -50.4 | 5.4 | 14.4 | 3.23 | 2e-03 |
| Planum Polare | left | -45.0 | -1.8 | -5.4 | 3.54 | 8e-04 |
| Parahippocampal Gyrus, anterior division | right | 32.4 | -10.8 | -25.2 | 3.37 | 1e-03 |
| Middle Temporal Gyrus, anterior division | left | -63.0 | 1.8 | -16.2 | 3.07 | 3e-03 |
| Middle Frontal Gyrus | right | 39.6 | 32.4 | 19.8 | 3.32 | 1e-03 |
| Lingual Gyrus | left | -7.2 | -81.0 | -18.0 | 3.94 | 3e-04 |
| | left | -23.4 | -46.8 | -7.2 | 3.6 | 7e-04 |
| | left | -12.6 | -55.8 | -10.8 | 2.66 | 7e-03 |
| Insular Cortex | left | -43.2 | -12.6 | 1.8 | 4.32 | 1e-04 |
| | right | 30.6 | 16.2 | 1.8 | 3.14 | 2e-03 |
| | left | -34.2 | -10.8 | -7.2 | 2.75 | 6e-03 |
| Inferior Temporal Gyrus, posterior division | left | -55.8 | -41.4 | -19.8 | 3.93 | 3e-04 |
| Inferior Frontal Gyrus, pars triangularis | right | 50.4 | 21.6 | 3.6 | 3.43 | 1e-03 |
| Frontal Pole | left | -28.8 | 55.8 | 18.0 | 3.94 | 3e-04 |
| | left | -12.6 | 61.2 | -7.2 | 3.94 | 3e-04 |
| | left | -27.0 | 52.2 | -18.0 | 3.41 | 1e-03 |
| Frontal Orbital Cortex | left | -18.0 | 27.0 | -12.6 | 4.04 | 2e-04 |
| | right | 39.6 | 19.8 | -9.0 | 4.0 | 3e-04 |
| | right | 27.0 | 21.6 | -25.2 | 3.85 | 4e-04 |
| | left | -46.8 | 27.0 | -12.6 | 3.48 | 1e-03 |
| | left | -21.6 | 18.0 | -21.6 | 3.08 | 3e-03 |
| Frontal Medial Cortex | right | 0.0 | 41.4 | -18.0 | 4.85 | 3e-05 |
| Cingulate Gyrus, anterior division | right | 7.2 | 28.8 | 12.6 | 3.31 | 1e-03 |
| | left | -3.6 | 32.4 | 7.2 | 3.23 | 2e-03 |
| Cerebral White Matter | left | -23.4 | 28.8 | 5.4 | 3.95 | 3e-04 |
| | right | 25.2 | 23.4 | 28.8 | 3.28 | 2e-03 |
| Cerebral Cortex | right | 12.6 | -75.6 | -27.0 | 4.01 | 3e-04 |
| Central Opercular Cortex | right | 54.0 | -3.6 | 5.4 | 4.96 | 2e-05 |
| | left | -52.2 | -7.2 | 10.8 | 4.17 | 2e-04 |
| Caudate | right | 16.2 | 16.2 | 12.6 | 3.66 | 6e-04 |
| Brain-Stem | left | -7.2 | -30.6 | -27.0 | 3.22 | 2e-03 |
| | right | 0.0 | -36.0 | -16.2 | 3.07 | 3e-03 |

Table 1: Brain areas within which decoding accuracy of the **Value** classifier was significantly ($p < 0.005$, minimum cluster extent threshold: 25 voxels) above chance, when classifier was trained on the **prox**. and tested on the **prox**. CS fractal. Brain labels are automatically generated according to the Harvard-Oxford atlas.

| Region | Hemisphere | x | y | z | t | p |
|---|---|---|---|---|---|---|
| Temporal Fusiform Cortex, anterior division | left | -41.4 | -5.4 | -25.2 | 4.14 | 2e-04 |
| Putamen | left | -27.0 | -12.6 | 9.0 | 4.31 | 1e-04 |
| Parahippocampal Gyrus, posterior division | right | 18.0 | -27.0 | -9.0 | 3.63 | 7e-04 |
| Paracingulate Gyrus | right | 10.8 | 41.4 | -1.8 | 3.95 | 3e-04 |
| | right | 12.6 | 45.0 | 10.8 | 2.73 | 6e-03 |
| Lingual Gyrus | left | -21.6 | -63.0 | -10.8 | 3.35 | 1e-03 |
| Insular Cortex | left | -39.6 | 5.4 | -3.6 | 3.56 | 8e-04 |
| | right | 28.8 | 10.8 | -12.6 | 2.79 | 5e-03 |
| Hippocampus | left | -23.4 | -34.2 | -7.2 | 3.64 | 7e-04 |
| Frontal Pole | right | 32.4 | 36.0 | 9.0 | 3.55 | 8e-04 |
| | right | 3.6 | 57.6 | -3.6 | 3.17 | 2e-03 |
| Cerebral White Matter | left | -16.2 | -12.6 | -10.8 | 5.25 | 1e-05 |
| | right | 28.8 | 21.6 | 19.8 | 3.47 | 1e-03 |
| | right | 32.4 | -1.8 | -12.6 | 3.22 | 2e-03 |
| Brain-Stem | left | -16.2 | -37.8 | -32.4 | 5.0 | 2e-05 |
| | right | 1.8 | -39.6 | -27.0 | 3.24 | 2e-03 |

Table 2: Brain areas within which decoding accuracy of the **Value** classifier was significantly ($p < 0.005$, minimum cluster extent threshold: 25 voxels) above chance, when classifier was trained on the **prox**. and tested on the **dist**. CS fractal. Brain labels are automatically generated according to the Harvard-Oxford atlas.

| Region | Hemisphere | x | y | z | t | p |
|---|---|---|---|---|---|---|
| Thalamus | right | 5.4 | -18.0 | 14.4 | 3.73 | 5e-04 |
| | right | 1.8 | -3.6 | 5.4 | 3.27 | 2e-03 |
| | right | 3.6 | -21.6 | 1.8 | 3.08 | 3e-03 |
| Temporal Pole | left | -52.2 | 18.0 | -10.8 | 4.15 | 2e-04 |
| | right | 50.4 | 19.8 | -14.4 | 2.74 | 6e-03 |
| Superior Temporal Gyrus, posterior division | right | 55.8 | -12.6 | -1.8 | 3.69 | 6e-04 |
| Putamen | left | -27.0 | 10.8 | -0.0 | 3.38 | 1e-03 |
| Pallidum | right | 27.0 | -9.0 | -3.6 | 3.43 | 1e-03 |
| Middle Frontal Gyrus | left | -34.2 | 36.0 | 23.4 | 3.11 | 2e-03 |
| Lingual Gyrus | right | 7.2 | -46.8 | -7.2 | 3.59 | 7e-04 |
| | left | -5.4 | -43.2 | -5.4 | 3.24 | 2e-03 |
| Insular Cortex | right | 36.0 | 19.8 | 5.4 | 3.32 | 1e-03 |
| Inferior Temporal Gyrus, temporooccipital part | left | -48.6 | -48.6 | -28.8 | 4.85 | 3e-05 |
| | right | 45.0 | -50.4 | -9.0 | 3.48 | 1e-03 |
| Inferior Frontal Gyrus, pars triangularis | right | 34.2 | 36.0 | 3.6 | 3.69 | 6e-04 |
| Frontal Pole | left | -50.4 | 37.8 | -10.8 | 4.53 | 7e-05 |
| | right | 0.0 | 66.6 | -0.0 | 4.05 | 2e-04 |
| | left | -34.2 | 39.6 | 7.2 | 4.04 | 2e-04 |
| | right | 5.4 | 61.2 | 25.2 | 3.36 | 1e-03 |
| | right | 46.8 | 36.0 | 5.4 | 2.86 | 4e-03 |
| Frontal Orbital Cortex | left | -34.2 | 30.6 | -23.4 | 4.78 | 4e-05 |
| | left | -21.6 | 32.4 | -18.0 | 3.17 | 2e-03 |
| Frontal Medial Cortex | right | 3.6 | 32.4 | -14.4 | 3.11 | 2e-03 |
| Cerebral White Matter | right | 23.4 | -3.6 | 18.0 | 3.81 | 4e-04 |
| Amygdala | left | -27.0 | -9.0 | -18.0 | 3.54 | 8e-04 |

Table 3: Brain areas within which decoding accuracy of the **Value** classifier was significantly ($p < 0.005$, minimum cluster extent threshold: 25 voxels) above chance, when classifier was trained on the **dist**. and tested on the **dist**. CS fractal. Brain labels are automatically generated according to the Harvard-Oxford atlas.

| Region | Hemisphere | x | y | z | t | p |
|---|---|---|---|---|---|---|
| Temporal Occipital Fusiform Cortex | left | -41.4 | -50.4 | -18.0 | 6.08 | 1e-06 |
| | left | -27.0 | -55.8 | -18.0 | 3.89 | 3e-04 |
| | right | 46.8 | -55.8 | -16.2 | 3.36 | 1e-03 |
| Occipital Fusiform Gyrus | left | -36.0 | -72.0 | -10.8 | 5.64 | 4e-06 |
| | right | 39.6 | -68.4 | -16.2 | 5.55 | 5e-06 |
| | right | 30.6 | -81.0 | -12.6 | 5.04 | 2e-05 |
| | left | -28.8 | -82.8 | -18.0 | 3.54 | 8e-04 |
| Middle Temporal Gyrus, posterior division | left | -61.2 | -28.8 | -18.0 | 4.81 | 3e-05 |
| Lingual Gyrus | left | -5.4 | -82.8 | -19.8 | 3.79 | 4e-04 |
| Insular Cortex | left | -28.8 | 27.0 | 7.2 | 3.62 | 7e-04 |
| | left | -37.8 | -7.2 | -7.2 | 3.55 | 8e-04 |
| Frontal Pole | left | -7.2 | 52.2 | -21.6 | 4.34 | 1e-04 |
| | right | 10.8 | 70.2 | 7.2 | 3.87 | 4e-04 |
| | right | 27.0 | 48.6 | -5.4 | 3.5 | 9e-04 |
| | left | -37.8 | 41.4 | 27.0 | 3.34 | 1e-03 |
| | right | 18.0 | 63.0 | 18.0 | 3.15 | 2e-03 |
| | left | -21.6 | 63.0 | -1.8 | 3.06 | 3e-03 |
| Cingulate Gyrus, anterior division | left | -7.2 | 32.4 | -1.8 | 3.24 | 2e-03 |
| Cerebral White Matter | right | 18.0 | 45.0 | 18.0 | 2.97 | 3e-03 |
| Brain-Stem | left | -3.6 | -21.6 | -16.2 | 3.64 | 7e-04 |

Table 4: Brain areas within which decoding accuracy of the **Identity** classifier was significantly ($p < 0.005$, minimum cluster extent threshold: 25 voxels) above chance, when classifier was trained on the **prox**. and tested on the **prox**. CS fractal. Brain labels are automatically generated according to the Harvard-Oxford atlas.

| Region | Hemisphere | x | y | z | t | p |
|---|---|---|---|---|---|---|
| Temporal Pole | left | -46.8 | 5.4 | -10.8 | 3.63 | 7e-04 |
| Superior Temporal Gyrus, posterior division | left | -55.8 | -18.0 | -1.8 | 4.15 | 2e-04 |
| Precentral Gyrus | left | -63.0 | 7.2 | 18.0 | 4.39 | 1e-04 |
| Lingual Gyrus | left | -21.6 | -61.2 | -7.2 | 3.31 | 1e-03 |
| Insular Cortex | right | 30.6 | 23.4 | 5.4 | 3.41 | 1e-03 |
| Inferior Frontal Gyrus, pars triangularis | right | 57.6 | 27.0 | 23.4 | 4.11 | 2e-04 |
| Frontal Pole | right | 32.4 | 37.8 | 9.0 | 4.07 | 2e-04 |
| | right | 34.2 | 37.8 | -18.0 | 4.0 | 3e-04 |
| | left | -37.8 | 48.6 | 14.4 | 3.62 | 7e-04 |
| | right | 1.8 | 59.4 | -14.4 | 3.14 | 2e-03 |
| Frontal Orbital Cortex | left | -16.2 | 14.4 | -23.4 | 4.2 | 2e-04 |
| | right | 19.8 | 14.4 | -16.2 | 3.73 | 5e-04 |
| * Cerebral Cortex | right | 9.0 | 5.4 | -12.6 | 3.53 | 9e-04 |
| | left | -23.4 | -57.6 | -21.6 | 3.0 | 3e-03 |
| Central Opercular Cortex | left | -43.2 | 7.2 | 10.8 | 4.77 | 4e-05 |
| Amygdala | right | 18.0 | -5.4 | -18.0 | 3.33 | 1e-03 |

Table 5: Brain areas within which decoding accuracy of the **Identity** classifier was significantly ($p < 0.005$, minimum cluster extent threshold: 25 voxels) above chance, when classifier was trained on the **prox**. and tested on the **dist**. CS fractal. Brain labels are automatically generated according to the Harvard-Oxford atlas. * Cerebral cortex label reflects labeling ambiguities in the Harvard-Oxford atlas.

| Region | Hemisphere | x | y | z | t | p |
|---|---|---|---|---|---|---|
| Temporal Pole | left | -55.8 | 16.2 | -14.4 | 2.84 | 5e-03 |
| Temporal Occipital Fusiform Cortex | right | 45.0 | -50.4 | -18.0 | 4.17 | 2e-04 |
| Subcallosal Cortex | left | -1.8 | 23.4 | -19.8 | 3.33 | 1e-03 |
| Putamen | right | 32.4 | -12.6 | -5.4 | 3.68 | 6e-04 |
| Planum Temporale | left | -54.0 | -28.8 | 5.4 | 3.04 | 3e-03 |
| Insular Cortex | left | -32.4 | 5.4 | -7.2 | 3.49 | 9e-04 |
| Inferior Frontal Gyrus, pars opercularis | right | 43.2 | 18.0 | 14.4 | 3.66 | 6e-04 |
| Hippocampus | left | -30.6 | -14.4 | -19.8 | 2.96 | 3e-03 |
| Heschl's Gyrus (includes H1 and H2) | right | 50.4 | -10.8 | 3.6 | 3.11 | 2e-03 |
| Frontal Pole | right | 52.2 | 34.2 | 19.8 | 3.74 | 5e-04 |
| Frontal Orbital Cortex | left | -27.0 | 32.4 | 1.8 | 4.18 | 2e-04 |
| Cerebral White Matter | right | 25.2 | 10.8 | 14.4 | 2.59 | 8e-03 |
| Caudate | right | 10.8 | 3.6 | 14.4 | 3.14 | 2e-03 |

Table 6: Brain areas within which decoding accuracy of the **Identity** classifier was significantly ($p < 0.005$, minimum cluster extent threshold: 25 voxels) above chance, when classifier was trained on the **dist**. and tested on the **dist**. CS fractal.
