# Supplementary material for: Evidence for model-based encoding of Pavlovian contingencies in the human brain
Source: Nat Commun. 2019 Mar 7;10:1099. doi: 10.1038/s41467-019-08922-7 (PMC6405831; doi:10.1038/s41467-019-08922-7)
Supplement: Supplementary file 2 — Peer Review [file 41467_2019_8922_MOESM2_ESM.pdf]

Reviewers' Comments:

Reviewer #1:
Remarks to the Author:
Pavlovian conditioning is generally considered a model-free mechanism, involving associations between predictive stimuli and the value of outcomes. This functional imaging study tests whether the human orbitofrontal cortex (OFC) and striatum represent model-based information (stimulus-stimulus associations) during Pavlovian conditioning. The clever design uses a sequence of two consecutive conditioned stimuli (CS, distal CS [CSd], proximal CS [CSp]) followed by juice or water as unconditioned stimulus (US). Pattern-based imaging analyses show that activity patters in the OFC encode not only the value of CS but also CSd-CSp identity associations. Moreover, CS-value and CSd-CSp identity associations were found in the ventral and dorsal striatum, respectively. However, decoding accuracies were conditional on behavioral markers of CS-value learning and explicit knowledge about the associative task structure, respectively. The results of this study provide new and important evidence for model-based representations in the OFC during Pavlovian conditioning, and suggest a dissociation between model-based and value representations in different areas of the striatum.

Overall this is a really nice contribution that provides novel and important findings. However, I have some reservations regarding the behavioral data and their presentation. In addition, it is not clear to me how exactly the decoding analyses were performed as the manuscript includes inconsistent statements. Some of these issues may affect the conclusions drawn from the results.

1. Are there any behavioral measures that could help support the notion that the current Pavlovian task involved model-based and/or model-free processes? I am not convinced that subjective value ratings for the CSd's provide are a good marker for either model-based or model-free processes. Those ratings could be based either on model-free second-order conditioning (CSd<-CSp<-US) or model-based inference (CSd->CSp->US). I do not see how we could dissociate between these two possibilities. If anything, the explicit knowledge test for CSd-CSp would support model-based processes, but these data need to be presented more clearly (see below).

2. The behavioral data should be presented using bar plots with error bars. It would be helpful to see average subjective value ratings for CSd+ and CSd- at pre and post, and average responses from the explicit knowledge test for CSd-CSp, CSd-US, and CSp-US associations. As mentioned above, explicit knowledge about CSd-CSp associations would provide support for the presence of model-based associations.

3. In addition, it would be informative to include (supplementary?) bar plots showing average pleasantness ratings for juice and water, for each of the four sessions. Are there any differential pleasantness changes for the two outcomes across sessions? Please also provide bar plots for hunger and thirst ratings.

4. The authors mention in the introduction and discussion section that stimulus-stimulus (CSd-CSp) associations in the current experiment are affectively neutral, and that this is what distinguishes their findings from previous results. However, CSd-CSp associations in the current experiment are not necessarily affectively neutral. By pairing the CSp with the US, the CSp will acquire model-free value and may act as conditioned reward to form value-based associations with the CSd. Thus, it is not clear from this experiment whether OFC really encodes value-neutral associations between two neutral stimuli. However, there is recent evidence in rodents suggesting that OFC indeed encodes value-neutral associations (Sadacca et al. 2018, eLIfe).

5. I found it difficult follow the logic of the MVPA analyses. In particular, it is unclear whether the different classifiers were trained and tested on activity corresponding to the CSd or CSp. There are conflicting statements regarding this issue throughout the manuscript. For instance, for the value classifier in the OFC the authors write "We trained and tested the classifier on patterns of activity elicited by presentation of the CSd fractal." However, when describing this classifier for the striatum, the authors state that "As with the OFC, we trained a classifier to decode the value of the CSp based on activity patterns present at the time of presentation of the CSp". Which of these two statements is correct? For both the OFC and striatum, was the value classifier trained on CSd or CSp activity? The legend for Figure 3 suggests that for the OFC, identity-based classifiers were trained on CSp activity and tested on CSd activity, whereas value-based classifiers were trained and tested on CSp activity. Is this correct? However, this would be inconsistent with the discussion which states that the value based classifier was tested on the distal cue ("we could decode whether the distal cue was associated with the subsequent delivery of the juice reward or the neutral non-rewarding liquid"). It is even less clear how the value- and identity-based classifiers were set up for the striatum. Were these analyses identical to the approach used in the OFC? This should be stated and clarified throughout the manuscript.

6. It is also not clear which behavioral measures were used for the correlation with decoding accuracy in the striatum. In particular, according to the legend in Figure 4c, identity decoding accuracy was correlated with the "explicit knowledge of the CS-US associations". Was this knowledge about CSd-US or CSp-US associations? More importantly, if the caudate body encodes model-based associations (CSd-CSp identity associations), shouldn't decoding accuracy correlate with knowledge about the CSd-CSp association, rather than CS-US association? Also, according to the discussion, decoding accuracies in the caudate were correlated with knowledge about stimulus-stimulus associations and not CS-US associations ("conditional on the degree to which a participant could report explicit knowledge of the stimulus-stimulus associations"). This is important for the interpretation of the caudate finding and should be clarified.

7. Did the authors test correlations between decoding accuracy for value in the ventral striatum and explicit knowledge about the CSd-CSp and CS-US associations?

8. New CSd-CSp associations were introduced each session. Is it a problem for the decoding analysis that learning occurs over the course of one session? The simulation provided in the supplementary materials suggests that this may account for the negative decoding accuracies. In this case, should we expect to obtain higher decoding accuracies if only the second half of trials per session was used?

9. I am also not sure I follow the across-subject cross-validation used for feature selection in the striatum. Parameters were optimized based on data from 85% of the subjects and then applied to the remaining 15% of subjects to train and test classifiers to obtain final decoding accuracies. Obviously, final decoding accuracies were obtained for all subjects, meaning that several cross-validation folds (85:15 splits) were implemented. Is this correct? How many cross-validation folds were used to obtain final decoding accuracies for all subjects? This should be clarified.

Reviewer #2:
Remarks to the Author:
The manuscript presented by Pauli et al. examines the nature of associative representations in orbitofrontal cortex and striatum in humans. The task involves presenting subjects with different chains of visual stimuli with a common associative structure. In each, the presentation of a distal stimulus reliably predicts presentation of a proximal stimulus, which in turn leads to presentation of

either a juice reward or neutral liquid. The structure of the task is such that subjects are encouraged to form a "cognitive map" of the model-based state transitions between the stimuli.

Evidence for model-based representations of the relationships between the stimuli can be isolated by revealing the decoding of identity of the upcoming visual stimuli. That is, being able to decode the identity of the upcoming proximal stimulus from activity elicited by presentation of the distal stimulus- irrespective of whether or not the stimulus has been paired with rewarding outcome- indicates the development of an associative map. This is in contrast to model-free representations of the stimulus relationships which would entail the backpropagation of value to the associated stimuli from the predicted rewarding outcome and be devoid of identity information.

Using mulitivoxel pattern analysis, Pauli et al. show that representations in orbitofrontal cortex encode information about the identity of the upcoming stimuli. Further, they demonstrate that evidence for these maps is also present in dorsal striatum (to the extent that participants are expressing explicit knowledge of the relationships between the stimuli). In contrast, activity in ventral striatum does not appear to reflect identity of the upcoming stimuli but, rather, the general value which has accrued to the stimulus as a result of being paired with the either the juice reward or neutral liquid (common across many stimulus chains).

The manuscript makes an interesting and novel addition to the literature and would be well suited to publication in Nature Communications. Below I have some general comments and requests for additional analyses that I think will make the paper even more appealing to readers. However, to be clear this is no way takes away from the novelty or informative nature of the results presented in the manuscript.

The first request I have is whether a trial-by-trial analysis may reveal the development of the associative structure of the task in orbitofrontal cortex and striatum. That is, does the correlation between the activity of the distal and proximal stimuli evolve as subjects receive more experience with the stimulus chains. This is particularly important given the temporal nature of the task. Specifically, as the stimuli are always presented consecutively in close succession, it is possible that the analysis revealing correlations between activity for the distal and proximal stimuli is confounded by normal variations in brain activity across time. Showing that these correlations evolve across experience with the task would alleviate this confound and stamp in the notion that these associations are the result of learning.

The second, related analysis that may add to the results is one that explores the nature of any interactions between orbitofrontal cortex and striatum. For example, if the associative representations of the task develop in the brain across trials, is there a difference in the rate at which this emerges across these regions? One might expect that the development of task-relevant activity in the orbitofrontal cortex precedes that of striatal regions. Further, is there a difference in how activity in these regions may be related depending on whether participants later reported accurate stimulus-stimulus relationships. That is, if representations in orbitofrontal cortex informs the development of cognitive maps in striatum, we might expect a causal relationship between activity in these regions when participants are accurately encoding the stimulus relationships. More in-depth analysis of the data may help to reveal a causal interaction of these circuits in learning and broaden our understanding of how associative knowledge guides behavior.

Finally, it is very clear to me that the hypothesis tested in the manuscript explicitly tests the theory put forwards by Wilson et al. (2016; Neuron). This theory presented by Wilson et al. (2016) argues that the orbitofrontal cortex represents a map of state space. More specifically, that the orbitofrontal cortex tracks movement through the state transitions associated with model-based reinforcement

learning. This is almost identical to the argument made by Pauli et al. in the current manuscript. While the Wilson et al. (2016) is cited in the discussion of the manuscript, I feel that it is warranted that this theory be mentioned in the introduction of the paper and the theory explicitly stated and explained. For example, on page 3 the statement: "A major open question remains despite the aforementioned evidence for the influence of MB computations on value signals and associated prediction errors: Where and how does the brain encode the cognitive map, or state-space transition model, needed for MB value computations during Pavlovian conditioning?" would be a prime opportunity to introduce the Wilson et al. (2016) model and it's predictions in relation to OFC (that may also now be extended to striatal regions). This would serve not to take away from the novelty of the paper but, rather, to better situate it within the current literature.

Minor comments:

- A recent manuscript explores the development of the similar associations in rat orbitofrontal cortex and should be cited and discussed accordingly (Sadacca et al., eLife 2018).

- On page 3, the authors mention that the associations between the distal and proximal fractals were permuted across sessions. Can the authors elaborate on what this means?

- More generally, I think the authors could report the task in more detail in the introduction of the manuscript.

- Is there a more intuitive way that the authors could represent the behavioral data?

Reviewer #3:
Remarks to the Author:
This paper reports results from multivoxel pattern analyses of data from 25 young volunteers, who performed a sequential Pavlovian conditioning paradigm, in which a 'distal' conditioned stimulus is followed by a 'proximal' conditioned stimulus which terminates in appetitive or neutral juice delivery. Analyses are focused on two regions of interest, the orbitofrontal cortex and the striatum, which are reported to 'encode a cognitive map of stimulus-stimulus associations alongside predictive value signals', in the striatum in a way that correlates with behavior. These findings are presented as overturning prominent accounts of Pavlovian conditioning which assume that such learning is exclusively model-free.

The experimental design, which allows disentangling of predictive representations of the identity and the value of a subsequent stimulus, is very clever; the study seems well conducted and the paper is well written. Moreover, the results nicely strengthen recent evidence for the encoding of predictive identity signals in the OFC at the time of an associated conditioned stimulus (Howard and Kahnt 2017). The finding that predictive signals are also seen in the striatum, in a regionally specific and behaviorally relevant manner is novel, will certainly stimulate thinking in the field and its interpretation is helped by the supplementary simulations.

Novelty:
What I wonder, however, is the degree to which the current findings represent a major or rather an incremental advance, relative to this prior work, in our mechanistic understanding of model-based computations in the OFC for Pavlovian conditioning. I have some difficulty appreciating the significance of the difference between that prior work and the current work in this context. The 'arbitrary [proximal] stimuli which are not potent reinforcers in their own right' in the current study

are secondary reinforcers. Can the authors specify more clearly and convincingly why exactly is it so important to extend this prior finding that the OFC encodes a predictive representation of the identity of primary reinforcement to that of secondary reinforcement? Apart from adding a more convincing clarification (or toning down the implied novelty of the OFC finding), to the paper's introduction, of the novel mechanistic questions addressed by the current study, I also recommend moving the description of this prior work to earlier in the introduction and removing the term 'preliminary' (or clarify why this prior evidence is preliminary). In the context of the novel observations in the striatum, I suggest the authors consider a paper by Bornstein and Daw in 2011 which highlights both model-based and model-free computations in the striatum for Pavlovian control.

Interpretation:
Is it adequate to refer to the finding of predictive representations of the identity of the proximal CS as evidence for a cognitive map or a state-space transition model? This might be more justified in the case of predictive representations of sequences of events. I would recommend staying closer to the actual findings and to refer to predictive identity representations (also in the title).

Statistical analyses:
It is unclear to me how valid it is, from the perspective of statistical independence, to test the accuracy of a classifier to decode proximal fractals at the time of the distal fractals when that classifier was trained to decode proximal fractals at the time of the proximal fractals that almost always immediately follow those distal fractals in a fixed order. Is this biased by potential mis-assigning of proximal CS-related variability to distal CS-related variability? In other words, can the authors reassure me that their key effects of interest are not biased statistically by the way that the classifiers were trained? I realize there is some jitter between CSd and CSp but this jittering does not seem particularly large, and the CSd event seems to stay on the screen until the CSp event. In the discussion, the authors refer to 'cross-decoding' in which one set of conditions was used for training and another for testing, but I cannot find reference to this or more details about this in the methods or results.

Moreover, I am confused by the description of the MVPA procedure in some places in the result section. For example, on page 10, the statement 'We trained and tested the classifier on patterns of activity elicited by presentation of the CSd fractal' as well as the legend to Figure 3. Doesn't this analysis (in Fig 3b) involve training the classifier to decode the value of proximal fractals using data at the time of the proximal fractals but testing this at the time of the distal fractals?

I recommend for Figure 3 applying either the threshold used for statistical inference, or unthresholded maps (with highlighting of significant effects). Also presentation of individual datapoints (as in 4b and 4c) for the OFC would be good for assessing consistency.

Multiple comparison correction (e.g. for the brain-behavior correlations) is better done for all comparisons across all ROIs, not just the number of comparisons within an ROI.

Regions of interest:
Given prior findings (e.g. by authors from the same lab), it would make sense to assess signals also in amygdala and/or hippocampus.

It is unclear to me to what end the meta-classifier was created, implementing a pipeline consisting of a PCA-based feature dimensionality reduction. Why not use the functional zones for parcellation as reported in the original paper?

Please present a figure showing image coverage.

Reviewer #1 (Remarks to the Author):

Pavlovian conditioning is generally considered a model-free mechanism, involving associations between predictive stimuli and the value of outcomes. This functional imaging study tests whether the human orbitofrontal cortex (OFC) and striatum represent model-based information (stimulus-stimulus associations) during Pavlovian conditioning. The clever design uses a sequence of two consecutive conditioned stimuli (CS, distal CS [CSd], proximal CS [CSp]) followed by juice or water as unconditioned stimulus (US). Pattern-based imaging analyses show that activity patterns in the OFC encode not only the value of CS but also CSd-CSp identity associations. Moreover, CS-value and CSd-CSp identity associations were found in the ventral and dorsal striatum, respectively. However, decoding accuracies were conditional on behavioral markers of CS-value learning and explicit knowledge about the associative task structure, respectively. The results of this study provide new and important evidence for model-based representations in the OFC during Pavlovian conditioning, and suggest a dissociation between model-based and value representations in different areas of the striatum.

Overall this is a really nice contribution that provides novel and important findings. However, I have some reservations regarding the behavioral data and their presentation. In addition, it is not clear to me how exactly the decoding analyses were performed as the manuscript includes inconsistent statements. Some of these issues may affect the conclusions drawn from the results.

1. Are there any behavioral measures that could help support the notion that the current Pavlovian task involved model-based and/or model-free processes?

The experiment was designed to obtain evidence for stimulus-stimulus representations in the brain that could be used to support model-based processing as opposed to providing behavioral evidence for model-based Pavlovian conditioning. In fact, behavioral evidence for model-based processing in Pavlovian conditioning is actually extensive, and for this reason we did not focus on demonstrating that here. The key evidence that Pavlovian conditioning exhibits properties that would be normally attributable to model-based processing is the fact that Pavlovian conditioning tends to be strongly devaluation sensitive -- that is conditioned responses such as auto-shaping to a Pavlovian CS are adjusted immediately following a chance in outcome value using devaluation procedures. See for example the detailed discussion of this point and review of relevant empirical studies by Berridge and Dayan (2013) that we reference in the manuscript.

I am not convinced that subjective value ratings for the CSd's provide are a good marker for either model-based or model-free processes. Those ratings could be based either on model-free second-order conditioning (CSd<-CSp<-US) or model-based inference (CSd->CSp->US). I do not see how we could dissociate between these two possibilities. If anything, the explicit knowledge test for CSd-CSp would support model-based processes, but these data need to be presented more clearly (see below).

We agree with the reviewer, that subjective value ratings could be the result of either MF or MB learning. We already touched on this issue in the discussion of the dissociation between the dorsal and the ventral striatum. Specifically, in the context of the correlation between the accuracy of the stimulus-value classifier and participants' subjective ratings, we discussed that our findings do not allow us to deliberate whether the ventral striatum encodes model-free or model-based value representations. We updated the manuscript to ensure that the reader does not draw the conclusion that changes in subjective stimulus ratings are necessarily indicative of model-free second-order conditioning. Specifically we added the following caveat to the Discussion on page 16:

"It is important to note that our results do not allow us to draw conclusions as to whether ventral striatal value signals are the result of model-based or model-free learning, as either or both mechanism could be responsible for the acquisition and expression of value signals in that region."

2. The behavioral data should be presented using bar plots with error bars. It would be helpful to see average subjective value ratings for CSd+ and CSd- at pre and post, and average responses from the explicit knowledge test for CSd-CSp, CSd-US, and CSp-US associations. As mentioned above, explicit knowledge about CSd-CSp associations would provide support for the presence of model-based associations.

We have added these additional figures to the supplementary figures (S2). We also now reference the supplementary figures in the caption of Figure 2. In response to a suggestion by another reviewer, we replaced the density plots of Figure 2 with violin plots, with individual data points, and boxplots overlay. The same style figure was used for the supplementary figures. We chose violin plots and boxplots over mean and variance, because the violin plots clearly show non-normality of the distribution. We updated the figure caption of the original Figure 2, to also mention there (in addition to the existing mention in the Methods section) that ratings were converted to a non-parametric measure, the number of rating changes contingent with the Pavlovian contingencies.

Interestingly, we found that while the overall test score for explicit knowledge of contingencies correlated with the performance of the stimulus-stimulus classifier in the dorsal striatum, post-hoc tests revealed that this correlation was carried predominantly by a correlation of classifier performance with both CSd-US and CSp-US test scores, but not with knowledge of CSd-CSp associations. We now report this finding in a supplementary figure and a paragraph in the discussion section of the manuscript. This finding represents evidence that the quality of model-based activity patterns in the dorsal striatum is especially associated with the ability of participants to remember CSp/d-US associations explicitly after the end of the final session. We think this result is interesting because it helps to link the stimulus-stimulus encoding found in the dorsal striatum more directly to model-based processing. The ultimate objective of a model-based Pavlovian system would be to establish which stimuli in the world are associated

with behaviorally relevant outcomes (whether a reward or non-reward). Model-based computations would therefore be expected to involve retrieval of the outcome features associated with particular stimulus chains in the environment, analogous to the process of "planning" in model-based instrumental conditioning. Thus, the fact that explicit knowledge reflects the identity of the outcome associated with particular stimuli, and that this depends on successful decoding of the interim stimulus-stimulus associations, supports a strong analogy between model-based inference in Pavlovian and instrumental case, and helps identify an important role for the dorsal striatum in the Pavlovian form of model-based inference. A consideration of these points is now added to the Discussion on page 17:

"A post-hoc analysis further revealed that stimulus classifier accuracy in the dorsal striatum was specifically correlated with stimulus-outcome knowledge. This finding suggests that the quality of model-based activity patterns in this region are associated with the ability of participants to remember stimulus-outcome associations explicitly after the end of the final session. This finding is important because a model-based Pavlovian agent ultimately needs to compute the value of different states of the world. To accomplish this it would be necessary to integrate knowledge of successive state-space transitions with knowledge about where in the state-space a rewarding outcome will be delivered. Here we found that the more discriminable representations of stimulus-stimulus associations were in the caudate nucleus, the better participants' explicit knowledge of the identity of the outcomes linked to those stimuli. This suggests the possibility that participants can actively utilize stimulus-stimulus knowledge in the dorsal striatum in order to determine states of the world ultimately lead to valuable as opposed to less valuable outcomes."

3. In addition, it would be informative to include (supplementary?) bar plots showing average pleasantness ratings for juice and water, for each of the four sessions. Are there any differential pleasantness changes for the two outcomes across sessions? Please also provide bar plots for hunger and thirst ratings.

We added these in supplementary Figure S4. Separate linear-mixed effects models for juice, water, hunger and thirst ratings revealed a statistically significant reduction of ratings of thirst, water and juice pleasantness, but not hunger, across sessions. These results are reported in the supplementary figure.

4. The authors mention in the introduction and discussion section that stimulus-stimulus (CSd-CSp) associations in the current experiment are affectively neutral, and that this is what distinguishes their findings from previous results. However, CSd-CSp associations in the current experiment are not necessarily affectively neutral. By pairing the CSp with the US, the CSp will acquire model-free value and may act as conditioned reward to form value-based associations with the CSd. Thus, it is not clear from this experiment whether OFC really encodes value-neutral associations between two neutral stimuli. However, there is recent evidence in

rodents suggesting that OFC indeed encodes value-neutral associations (Sadacca et al. 2018, eLIfe).

We completely agree that CSd-CSp associations are not affectively neutral in the current experiment. However, because of our choice of cross-validation procedure for training and testing the classifier, in combination with the reversal of the value of each of the two CSp fractals (X and Y) in between each session of Pavlovian conditioning (such that each CSp is affectively positive in one half of sessions and affectively neutral in the other half of sessions), a machine learning classifier trained to distinguish between these two CSp fractals will succeed only if it ignores the affective value of a CSp fractal on a particular session, and focuses instead on the identity of the CS. Put another way, in each cross validation fold, each CSp is affectively neutral in one half of trials and affectively positive in the other, so that the affective quality balances out across the sessions and does not benefit the classification of the fractal identity. We have updated the manuscript to clarify this argument.

For instance in the introduction we now write (on page 4):
"Even though we anticipate that the CSp fractals will acquire affective value through learning within each session, in order to correctly identify the identity of the CSp fractal, affective information has to be ignored by the classifier, because in half of the sessions of both training and test sets, the affective value of a proximal CS fractal will be positive, while it will be neutral in the remaining half. If Pavlovian conditioning in humans does not invoke MB computations, such a classifier would not be able to successfully decode the identity of the proximal stimulus. When testing for cognitive map representations in the human brain, we focused on two brain regions: the striatum and the OFC."

5. I found it difficult follow the logic of the MVPA analyses. In particular, it is unclear whether the different classifiers were trained and tested on activity corresponding to the CSd or CSp. There are conflicting statements regarding this issue throughout the manuscript. For instance, for the value classifier in the OFC the authors write "We trained and tested the classifier on patterns of activity elicited by presentation of the CSd fractal." However, when describing this classifier for the striatum, the authors state that "As with the OFC, we trained a classifier to decode the value of the CSp based on activity patterns present at the time of presentation of the CSp". Which of these two statements is correct? For both the OFC and striatum, was the value classifier trained on CSd or CSp activity? The legend for Figure 3 suggests that for the OFC, identity-based classifiers were trained on CSp activity and tested on CSd activity, whereas value-based classifiers were trained and tested on CSp activity. Is this correct? However, this would be inconsistent with the discussion which states that the value based classifier was tested on the distal cue ("we could decode whether the distal cue was associated with the subsequent delivery of the juice reward or the neutral non-rewarding liquid"). It is even less clear how the value- and identity-based classifiers were set up for the striatum. Were these analyses identical to the approach used in the OFC? This should be stated and clarified throughout the manuscript.

As the reviewer correctly states, in the OFC we trained and tested the value classifier on BOLD responses to the distal CS fractal. In contrast, the identity classifier was trained on the OFC BOLD responses to the proximal CS fractal, and tested on the distal CS fractal. In the striatum, either classifier was trained on the proximal CS fractal and tested on the distal CS fractal. Thus, the training regimen for both classifiers in the striatum is identical to the training regimen for the identity classifier in the OFC. This is now clarified in the text.

For the OFC, we chose to focus on the result for training and testing the value classifier on the distal CS fractal, instead of the results of training on proximal and testing on distal CS fractals. We trained and tested the classifier on patterns of activity elicited by presentation of the CSd fractal, because this stimulus was the earliest predictor of reward delivery, hence relating these results to previous studies with canonical Pavlovian conditioning paradigms. However, as shown in supplementary Tables S1-3, we do find value representations on the orbital surface (extending to the ventral frontal pole) irrespective of whether training is implemented on the distal or proximal training schedule.

6. It is also not clear which behavioral measures were used for the correlation with decoding accuracy in the striatum. In particular, according to the legend in Figure 4c, identity decoding accuracy was correlated with the "explicit knowledge of the CS-US associations". Was this knowledge about CSd-US or CSp-US associations? More importantly, if the caudate body encodes model-based associations (CSd-CSp identity associations), shouldn't decoding accuracy correlate with knowledge about the CSd-CSp association, rather than CS-US association? Also, according to the discussion, decoding accuracies in the caudate were correlated with knowledge about stimulus-stimulus associations and not CS-US associations ("conditional on the degree to which a participant could report explicit knowledge of the stimulus-stimulus associations"). This is important for the interpretation of the caudate finding and should be clarified.

As we mentioned above in response to comment #2 by the reviewer, we did find that there was an overall correlation between the test score for explicit knowledge of Pavlovian contingencies (including S-0 and S-S associations) and stimulus classifier accuracy in the caudate. However, on closer inspection we found that this correlation was mainly driven by a correlation between classifier accuracy and test score for either CSd-US ($r=.55$, $p=0.004$) and CSp-US ($r=.56$, $p=0.003$) knowledge.

7. Did the authors test correlations between decoding accuracy for value in the ventral striatum and explicit knowledge about the CSd-CSp and CS-US associations?

Yes we did, but did not find any such correlation. This is now more clearly stated in the manuscript.

8. New CSd-CSp associations were introduced each session. Is it a problem for the decoding analysis that learning occurs over the course of one session? The simulation provided in the supplementary materials suggests that this may account for the negative decoding accuracies. In this case, should we expect to obtain higher decoding accuracies if only the second half of trials per session was used?

It's certainly possible that effects of learning will impact early trials in a session. However, unfortunately it is difficult for us to test for differential decoding accuracies in later vs earlier trials because the reduced statistical power that would follow from a marked reduction of the number of training samples (trials/CS onsets) would offset any potential gains in accuracy that might arise from testing later in the session.

9. I am also not sure I follow the across-subject cross-validation used for feature selection in the striatum. Parameters were optimized based on data from 85% of the subjects and then applied to the remaining 15% of subjects to train and test classifiers to obtain final decoding accuracies. Obviously, final decoding accuracies were obtained for all subjects, meaning that several cross-validation folds (85:15 splits) were implemented. Is this correct? How many cross-validation folds were used to obtain final decoding accuracies for all subjects? This should be clarified.

We updated the manuscript to clarify this point. We created 1000 cross-validation splits, in each split we selected 85% participants (without replacement) for parameter tuning and classifier training, and 15% for testing. This was done because we found that results were unstable across repeated analysis runs, if we e.g. created 5 * 80/20 splits, or 10 * 90/10 splits.

Reviewer #2 (Remarks to the Author):

The manuscript presented by Pauli et al. examines the nature of associative representations in orbitofrontal cortex and striatum in humans. The task involves presenting subjects with different chains of visual stimuli with a common associative structure. In each, the presentation of a distal stimulus reliably predicts presentation of a proximal stimulus, which in turn leads to presentation of either a juice reward or neutral liquid. The structure of the task is such that subjects are encouraged to form a "cognitive map" of the model-based state transitions between the stimuli.

Evidence for model-based representations of the relationships between the stimuli can be isolated by revealing the decoding of identity of the upcoming visual stimuli. That is, being able to decode the identity of the upcoming proximal stimulus from activity elicited by presentation of

the distal stimulus- irrespective of whether or not the stimulus has been paired with rewarding outcome- indicates the development of an associative map. This is in contrast to model-free representations of the stimulus relationships which would entail the backpropagation of value to the associated stimuli from the predicted rewarding outcome and be devoid of identity information.

Using mulitivoxel pattern analysis, Pauli et al. show that representations in orbitofrontal cortex encode information about the identity of the upcoming stimuli. Further, they demonstrate that evidence for these maps is also present in dorsal striatum (to the extent that participants are expressing explicit knowledge of the relationships between the stimuli). In contrast, activity in ventral striatum does not appear to reflect identity of the upcoming stimuli but, rather, the general value which has accrued to the stimulus as a result of being paired with the either the juice reward or neutral liquid (common across many stimulus chains).

The manuscript makes an interesting and novel addition to the literature and would be well suited to publication in Nature Communications. Below I have some general comments and requests for additional analyses that I think will make the paper even more appealing to readers. However, to be clear this is no way takes away from the novelty or informative nature of the results presented in the manuscript.

1. The first request I have is whether a trial-by-trial analysis may reveal the development of the associative structure of the task in orbitofrontal cortex and striatum. That is, does the correlation between the activity of the distal and proximal stimuli evolve as subjects receive more experience with the stimulus chains. This is particularly important given the temporal nature of the task. Specifically, as the stimuli are always presented consecutively in close succession, it is possible that the analysis revealing correlations between activity for the distal and proximal stimuli is confounded by normal variations in brain activity across time. Showing that these correlations evolve across experience with the task would alleviate this confound and stamp in the notion that these associations are the result of learning.

We agree that showing that these representations evolve over the course of training would be powerful. However, unfortunately we do not think this approach will be feasible based on the current task and dataset, because the current task is simply not designed, nor optimized, for trial-by-trial analysis. We appreciate the reviewer's concern regarding the potential for confounding effects due to the temporal nature of this task. However, we carefully chose our cross-validation approach to rule out this possibility. Importantly, we did not train the classifier on the proximal CS and test the classifier on the distal CS of the SAME trials (or session). Indeed, had we done that, there is the risk of successful decoding from the two time points being induced by auto-correlations that the reviewer rightly is concerned about. However, to avoid this pitfall, we trained our classifier on BOLD responses to proximal CS fractals from two sessions, and then tested its performance in decoding BOLD responses to distal CS fractals of two held-out sessions, thereby ensuring decoding was done on completely separate trials to that used for training. Thus, we ensured that auto-correlations within trials can be ruled as

driving classifier performance. In fact, we can confirm that the reviewer's concern would be justified if we had not done this in-between session cross-validation: In a separate analysis, we had implemented odd vs even trial cross-validation, and a permutation test indeed revealed that classifier performance was artifactually above chance due to non-independence of the training and test data in that case. However, in the case of the cross-validated session-based decoding analysis we reported in the manuscript, the permutation test revealed no such artifactual bias in classifier accuracy, indicating that our decoding strategy was appropriately unbiased. Furthermore, to rule out that successful decoding at the time of the distal stimulus is driven by artifactual leakage of the BOLD signal from the proximal stimulus, we examined the correlation between onset regressors set at the time of the distal stimulus convolved by a canonical hemodynamic response function, and onset regressors set at the time of the proximal stimulus also convolved with a HRF. If leakage could explain the decoding accuracies, we would expect a high correlation between the regressors set at these two time points. Alternatively, if the experimental design successfully decoupled these BOLD responses at these two time points we would expect a low correlation. As reported in a new supplementary figure S6, the average correlation between regressors at these time points was strikingly low at 0.13 (median), implying a very low shared variance (<1.7%) between these regressors. This helps to rule out the explanation for our findings as being due to leakage between proximal and distal stimuli.

We also updated the Methods section to clearly explain this reasoning for our choice of statistical analyses and cross-validation.

2. The second, related analysis that may add to the results is one that explores the nature of any interactions between orbitofrontal cortex and striatum. For example, if the associative representations of the task develop in the brain across trials, is there a difference in the rate at which this emerges across these regions? One might expect that the development of task-relevant activity in the orbitofrontal cortex precedes that of striatal regions. Further, is there a difference in how activity in these regions may be related depending on whether participants later reported accurate stimulus-stimulus relationships. That is, if representations in orbitofrontal cortex informs the development of cognitive maps in striatum, we might expect a causal relationship between activity in these regions when participants are accurately encoding the stimulus relationships. More in-depth analysis of the data may help to reveal a causal interaction of these circuits in learning and broaden our understanding of how associative knowledge guides behavior.

We agree that this would be an interesting question to investigate. However, the task design in combination with the MVPA methodology is not optimized for allowing us to answer this question. The reason is that this would require a trial-based decoding analysis, and we would need sufficient numbers of trials to train the classifier and subsequently test it for each timepoint or phase in the session. Given we have only 4 sessions, there is not enough training and test data to enable us to divide up the sessions in this way. Another approach to this would be try single-trial testing and decoding (using a leave-one trial out approach or training and testing on odd or even trials). However, when we tested a classifier based on this approach we found that

auto-correlations and inter-dependencies between trials violated the statistical properties of the MVPA approach -- this would be highly problematical as training and test data sets would end up being correlated, giving rise to an artifactual decoding success. The cross session decoding strategy we do use is not susceptible to this bias as discussed in the Supplementary Methods. Perhaps this could be achieved in a follow on experiment if were to obtain substantially more data from each individual subject, for instance by scanning each individual repeatedly on multiple days, so as to obtain sufficient numbers of sessions to enable such a decoding strategy, with appropriate cross-session decoding. We do however acknowledge that this as an important future direction in the Discussion.

Now added to the Discussion on page 21:

“Along these lines, an important direction for future work would be to attempt to characterize the relative contributions of these areas as a function of time, both within trials and across trials as a function of Pavlovian learning.”

3. Finally, it is very clear to me that the hypothesis tested in the manuscript explicitly tests the theory put forwards by Wilson et al. (2016; Neuron). This theory presented by Wilson et al. (2016) argues that the orbitofrontal cortex represents a map of state space. More specifically, that the orbitofrontal cortex tracks movement through the state transitions associated with model-based reinforcement learning. This is almost identical to the argument made by Pauli et al. in the current manuscript. While the Wilson et al. (2016) is cited in the discussion of the manuscript, I feel that it is warranted that this theory be mentioned in the introduction of the paper and the theory explicitly stated and explained. For example, on page 3 the statement: “A major open question remains despite the aforementioned evidence for the influence of MB computations on value signals and associated prediction errors: Where and how does the brain encode the cognitive map, or state-space transition model, needed for MB value computations during Pavlovian conditioning?” would be a prime opportunity to introduce the Wilson et al. (2016) model and it’s predictions in relation to OFC (that may also now be extended to striatal regions). This would serve not to take away from the novelty of the paper but, rather, to better situate it within the current literature.

We agree and updated the introduction to also introduce Wilson et al.’s (2016) model. This is done on page 6 of the introduction:

“Indeed, a prominent theoretical proposition suggests a role for the OFC in encoding a flexible cognitive map (Wilson et al., 2016).”

Minor comments:

- *A recent manuscript explores the development of the similar associations in rat orbitofrontal cortex and should be cited and discussed accordingly (Sadacca et al., eLife 2018).*

We now report this finding in our discussion section.

- *On page 3, the authors mention that the associations between the distal and proximal fractals were permuted across sessions. Can the authors elaborate on what this means?*

We appreciate this comment, as “permuted” is not the most appropriate term here. We meant to say that we reversed the value of each of the two CSp fractals (X and Y) in between session of Pavlovian conditioning (such that each CSp is affectively positive in one half of sessions and affectively negative in the other half of sessions). So that two orthogonal classifiers could be trained, one on classifying fractals based on their identity (X vs. Y), and one based on affective value (CS+ vs. CS-). We updated this paragraph of the manuscript to better describe this procedure.

- *More generally, I think the authors could report the task in more detail in the introduction of the manuscript.*

We now added substantially more details of the task to the introduction. We also mention in the Method section that additional details can be found in a previous study by our lab (Pauli et al., 2015).

- *Is there a more intuitive way that the authors could represent the behavioral data?*

We updated figure 2, using violin plots, which we believe to be both more intuitive and informative.

Reviewer #3 (Remarks to the Author):

This paper reports results from multivoxel pattern analyses of data from 25 young volunteers, who performed a sequential Pavlovian conditioning paradigm, in which a ‘distal’ conditioned stimulus is followed by a ‘proximal’ conditioned stimulus which terminates in appetitive or neutral juice delivery. Analyses are focused on two regions of interest, the orbitofrontal cortex and the striatum, which are reported to ’encode a cognitive map of stimulus-stimulus associations alongside predictive value signals’, in the striatum in a way that correlates with behavior. These findings are presented as overturning prominent accounts of Pavlovian conditioning which assume that such learning is exclusively model-free.

The experimental design, which allows disentangling of predictive representations of the identity and the value of a subsequent stimulus, is very clever; the study seems well conducted and the paper is well written. Moreover, the results nicely strengthen recent evidence for the encoding of

predictive identity signals in the OFC at the time of an associated conditioned stimulus (Howard and Kahnt 2017). The finding that predictive signals are also seen in the striatum, in a regionally specific and behaviorally relevant manner is novel, will certainly stimulate thinking in the field and its interpretation is helped by the supplementary simulations.

Novelty:
What I wonder, however, is the degree to which the current findings represent a major or rather an incremental advance, relative to this prior work, in our mechanistic understanding of model-based computations in the OFC for Pavlovian conditioning. I have some difficulty appreciating the significance of the difference between that prior work and the current work in this context. The 'arbitrary [proximal] stimuli which are not potent reinforcers in their own right' in the current study are secondary reinforcers. Can the authors specify more clearly and convincingly why exactly is it so important to extend this prior finding that the OFC encodes a predictive representation of the identity of primary reinforcement to that of secondary reinforcement?

We strove to clarify the novelty of the presented findings in the present version of the manuscript. The rationale is that in order to get evidence for a flexible encoding of a state-space transition structure, it is important to show that predictive stimulus identity coding can occur even with stimuli that have no innate value or interest to the animal (except by virtue of what those sequential stimuli ultimately predict). Studies showing that a CS can retrieve US identity in the OFC for instance are of course highly informative, but such studies could still leave open the possibility that OFC is involved in a more rigid type of associative learning only between stimuli and primary reinforcers, without necessarily playing a more general role in encoding a flexible cognitive map of stimulus-stimulus associations. Given that we know the OFC represents the value of different kinds of primary reinforcers such as sweet or unpleasant tastes, or food-related or disgust-related odors etc., one possibility could be that the OFC is exclusively involved in facilitating associations to be learned between arbitrary stimuli and those primary reinforcers, and that a more flexible coding encoding of state-space could occur instead elsewhere in the brain. We think this possibility is unlikely, but still, in order to rule this out it is important to show that the OFC implements a more flexible kind of stimulus-stimulus encoding, not involving a US that could arguably have "innate" or at least a long-standing (over the course of a lifetime of experience) acquired value.

This point is clarified further in the introduction on page 7:

"In order to establish whether the OFC is involved in encoding a more general and flexible cognitive map as opposed to exclusively mediating the learning of associations between arbitrary stimuli and affectively significant stimuli, it is necessary to demonstrate that the OFC encodes relationships between arbitrary stimuli which are not potent reinforcers in their own right. Thus, in the present study we aimed to test whether OFC encodes the identity of stimuli which have been arbitrarily associated with other stimuli without preexisting affective

significance, which would be consistent with the encoding of a flexible cognitive map of stimulus-stimulus associations."

Nevertheless we have also toned down the strength of our claims about the OFC for the reason that when plotting the mean accuracies from the OFC clusters following the request of the reviewer (now shown in Fig 4c), we found that the mean decoding accuracies were quite close to chance levels (even though the group statistics were statistically significant, implying that while the accuracies are low they are relatively consistent across participants). For this we added a caveat about these results to the Discussion. We also now focus the manuscript more on the striatal results which we believe to be more definitive because of the clear link between striatal decoding accuracy and behavior.

Apart from adding a more convincing clarification (or toning down the implied novelty of the OFC finding), to the paper's introduction, of the novel mechanistic questions addressed by the current study, I also recommend moving the description of this prior work to earlier in the introduction and removing the term 'preliminary' (or clarify why this prior evidence is preliminary). In the context of the novel observations in the striatum, I suggest the authors consider a paper by Bornstein and Daw in 2011 which highlights both model-based and model-free computations in the striatum for Pavlovian control.

Our findings also distinguish themselves from previous findings in that we found evidence in the central OFC and the dorsal striatum for representations of the identity of anticipated CS fractals, such that these representations persisted despite changes in the affective value of these CS fractals across sessions of Pavlovian conditioning.

We added a reference to the opinion paper by Bornstein and Daw (2011) to the introduction, next to the Neuron paper by Daw et al. (2011) presenting original results on model-based computations in the striatum.

We agree that the term "preliminary evidence" was not an ideal choice of words. We replaced it with "some existing evidence".

Interpretation:
Is it adequate to refer to the finding of predictive representations of the identity of the proximal CS as evidence for a cognitive map or a state-space transition model? This might be more justified in the case of predictive representations of sequences of events. I would recommend staying closer to the actual findings and to refer to predictive identity representations (also in the title).

Our results do show that participants can anticipate future states in a sequence of events leading up to reward in a Pavlovian conditioning. The ability to anticipate a future state on the basis of a probabilistic or other form of association with previous states, is in our view, at the

core of what is meant by a cognitive map. Our experiment was specifically designed to enable us to detect these forms of associations, hence the use of the term cognitive map. However, given that others may use the term cognitive map differently, we agree to remove this term from the title, and while occasionally continuing to refer to the term “cognitive map” we are now more clear throughout the manuscript that we are referring specifically to the ability to predict subsequent states.

Statistical analyses:

It is unclear to me how valid it is, from the perspective of statistical independence, to test the accuracy of a classifier to decode proximal fractals at the time of the distal fractals when that classifier was trained to decode proximal fractals at the time of the proximal fractals that almost always immediately follow those distal fractals in a fixed order. Is this biased by potential mis-assigning of proximal CS-related variability to distal CS-related variability? In other words, can the authors reassure me that their key effects of interest are not biased statistically by the way that the classifiers were trained? I realize there is some jitter between CSd and CSp but this jittering does not seem particularly large, and the CSd event seems to stay on the screen until the CSp event. In the discussion, the authors refer to ‘cross-decoding’ in which one set of conditions was used for training and another for testing, but I cannot find reference to this or more details about this in the methods or results.

We appreciate the reviewer’s justified concern regarding the potential for confounding effects due to the temporal nature of this task. We also addressed this point in response to reviewer #1. For convenience we recapitulate this point here. There are several aspects of our experiment and approach that minimize this possibility: Firstly, we carefully chose our cross-validation approach to rule out auto-correlations between the training and test data. Specifically, we trained a classifier on BOLD responses to proximal CS fractals from two of the four sessions, and then tested its performance in decoding BOLD responses to distal CS fractals from the remaining held out sessions. This helps to ensure that non-specific temporal auto-correlations can be ruled out as driving classifier performance. In fact, we can confirm that the reviewer’s concern would be justified if we had not done this in-between session cross-validation: In a separate analysis, we had done odd-even trial cross-validation, and a permutation test indeed revealed that classifier performance was above chance. We updated the Methods section to clearly explain the reasoning for our choice of statistical analyses and cross-validation. Secondly, we designed our experiment to ensure minimum correlations between the evoked BOLD responses at the time of the distal and proximal stimulus. We now report the distribution of these correlations across subjects and sessions in Figure S6. As shown in this figure, the average correlation between regressors at the time of the distal and the time of the proximal stimulus (after convolution with a hemodynamic response function) was 0.13 (median), implying a very low shared variance (<1.7%) between these regressors. This means that it is very unlikely that classifier decoding performance at the time of the distal cue can be explained by artifactual leakage of BOLD signals from the proximal cue.

We also appreciate the reviewer's observation regarding a missing reference to the 'cross-decoding' procedure. The incorrect study out of two "Haynes & Rees (2005)" studies had originally been included in the bibliography. This has been corrected.

Moreover, I am confused by the description of the MVPA procedure in some places in the result section. For example, on page 10, the statement 'We trained and tested the classifier on patterns of activity elicited by presentation of the CSd fractal' as well as the legend to Figure 3. Doesn't this analysis (in Fig 3b) involve training the classifier to decode the value of proximal fractals using data at the time of the proximal fractals but testing this at the time of the distal fractals?

I recommend for Figure 3 applying either the threshold used for statistical inference, or unthresholded maps (with highlighting of significant effects). Also presentation of individual datapoints (as in 4b and 4c) for the OFC would be good for assessing consistency.

We updated the caption of panel b of the OFC Figure, as the reviewer points out correctly, these results are indeed from training and testing the classifier on BOLD responses to the distal CS, rather than the proximal CS fractal .

We left the threshold of statistical maps at $p<0.005$, because this is indeed the threshold we applied in our brain behavior correlation analyses (see subsection "Brain behavior correlations" in the "Methods" section). That is the present threshold provides more accurate information about which voxels were included in the brain behavior correlation analyses. While we report that these results also survive correction for multiple comparisons (using FDR and TFCE), we don't see a need for separate figures.

We added individual accuracies for the cluster of the reward and the identify classifiers to the figure. Note as mentioned earlier, after creating this figure we noticed that the average decoding accuracies were quite low for both of the OFC decoding classifiers, even though they are statistically significantly above chance. Even though it is has been pointed out that decoding accuracies are often just above chance in frontal regions (Bhandari et al, 2018), we believe it is appropriate for us to acknowledge that the OFC results will need subsequent replication in order to increase confidence in their robustness, a caveat we now add to the Discussion. Also as mentioned earlier, we believe that the striatum results are more definitive because of the correlations we found with behavior that did survive multiple comparison correction across the ROIs. For this reason, we switched the order of the results in the Results section so that we present the more definitive striatal results first, and the orbitofrontal cortex findings second, so that the reader can view our findings in order of the relative strength of the conclusions we can draw. Thus, what was previously Figure 3 is now Figure 4 in the manuscript, and vice versa.

Multiple comparison correction (e.g. for the brain-behavior correlations) is better done for all comparisons across all ROIs, not just the number of comparisons within an ROI.

We did in fact correct for the number of ROIs, rather than for the number of comparisons within an ROI. All effects would also survive correction for multiple comparisons that additionally take into account that we ran two separate correlation analyses.

Regions of interest:
Given prior findings (e.g. by authors from the same lab), it would make sense to assess signals also in amygdala and/or hippocampus.

We agree that these are also regions of interest, but they were not the main focus of the present study. We had therefore chosen to only present these results in supplementary tables S1-S6.

It is unclear to me to what end the meta-classifier was created, implementing a pipeline consisting of a PCA-based feature dimensionality reduction. Why not use the functional zones for parcellation as reported in the original paper?

We appreciate this comment and strove to clarify our reasoning for this choice in the Methods. In short, in order to increase the signal to noise ratio and minimize the risk of overfitting, we chose to perform dimensionality reduction to achieve a more reasonable ratio of features to samples, as it is well documented that for reasonable classifier performance it is important that the proportion of features are substantially less than the amount of samples (Hastie, Tibshirani, & Friedman, 2009, chapter 7).

Please present a figure showing image coverage.

We now added supplementary figure S5 to show coverage.

Reviewers' Comments:

Reviewer #1:
Remarks to the Author:
The authors have fully addressed my initial comments and concerns. In particular I found the clarifications regarding the MVPA methods helpful and convincing.

I have only one additional comment. The authors may want to discuss their findings in the context of a recent paper by Howard and Kahnt (2018, Nature Communications). This study shows how CS->US-identity associations in the OFC change with learning, and there is a suggestion that these associations are updated through dopaminergic error signals in the midbrain. Is it possible that the model-based associations the authors found in the striatum are driven by similar error signals?

Reviewer #2:
Remarks to the Author:
The authors have addressed my concerns and the manuscript is suitable for publication in Nature Communications.

Reviewer #3:
Remarks to the Author:
The authors have addressed various of the comments and the revisions are appreciated. I like the paper, but have the following residual remarks:

I can see the added value of the present paper. Nevertheless, I would like to reiterate my recommendation of moving reference to the Howard et al 2015 (PNAS) paper to earlier in the introduction, e.g. following the reference to the Prevost work on page 3 of the introduction (please be reassured that there is no conflict of interest here). Also, please remove the word 'some' when referring to this existing evidence on page 6 of the introduction.

The rationale for distinct decoding strategies for OFC and striatum is unconvincing. Why not apply the same strategy for both regions?

I am left confused by the justification of the specific cross-subject cross-validation procedure in the rebuttal of R1's comment #9. What are the implications of the observation that "results were unstable across repeated analysis runs, if we e.g. created 5 * 80/20 splits or 10 * 90/10 splits." Should we be concerned about this?

It is not so clear that there is not enough power to assess learning-related effects by comparing e.g. first vs second half of the trials per session. Please elaborate.

It is unclear how the cross-validation procedure, in which the classifier was trained on different sessions than the sessions on which it was tested, address the question whether the temporal nature of the task (i.e. presentation of distal and proximal CSs in close succession) introduced confound. The finding that there is little shared variance between the distal and proximal CS is reassuring, but nevertheless I would suggest to add this issue as a caveat that is hard to fully rule out in the discussion.

Are the data presented here the same as those presented in your 2015 paper?

I generally like to see the actual changes implemented in the ms reiterated in the rebuttal letter. For next time.

Reviewers' comments:

Reviewer #1 (Remarks to the Author):

The authors have fully addressed my initial comments and concerns. In particular I found the clarifications regarding the MVPA methods helpful and convincing.

I have only one additional comment. The authors may want to discuss their findings in the context of a recent paper by Howard and Kahnt (2018, Nature Communications). This study shows how CS->US-identity associations in the OFC change with learning, and there is a suggestion that these associations are updated through dopaminergic error signals in the midbrain. Is it possible that the model-based associations the authors found in the striatum are driven by similar error signals?

We agree that there is accumulating evidence that interactions between the OFC and the dopamine system appear to be critically involved in the acquisition of identity outcome representations, including the evidence reported in the recent Howard and Kahnt study (2018).

We now mention these findings in the Conclusions section of the manuscript (Page 19, paragraph 2): “Recent evidence suggests that the formation of these anticipatory representations of unconditioned stimulus identity may be supported by identity-based error signals in the midbrain (Howard & Kahnt, 2018).”

Reviewer #2 (Remarks to the Author):

The authors have addressed my concerns and the manuscript is suitable for publication in Nature Communications.

Reviewer #3 (Remarks to the Author):

The authors have addressed various of the comments and the revisions are appreciated. I like the paper, but have the following residual remarks:

I can see the added value of the present paper. Nevertheless, I would like to reiterate my recommendation of moving reference to the Howard et al 2015 (PNAS) paper to earlier in the introduction, e.g. following the reference to the Prevost work on page 3 of the introduction (please be reassured that there is no conflict of interest here). Also, please remove the word ‘some’ when referring to this existing evidence on page 6 of the introduction.

We have added a reference to Howard et al. (2015) to the suggested location and removed the word ‘some’.

The rationale for distinct decoding strategies for OFC and striatum is unconvincing. Why not apply the same strategy for both regions?

Note that we did train and test from the same timepoints in the OFC as in the striatum (every possible permutation of the decoding strategy for OFC is reported in the supplementary tables).

Another way in which the OFC and striatum differed is in relation to the use of a searchlight vs ROI-based analysis in the OFC and striatum respectively. Perhaps this is what the reviewer is referring to.

To address the comment, we re-worded the relevant parts of the Results section to explain our rationale more clearly (page 10, paragraph 2):

“*We determined that the spherical searchlight procedure we used for cortical analyses (including OFC) is not as appropriate for sub-cortical structures such as the striatum. The reason is that a number of neuranatomically and functionally distinct regions (including ventricles) are tightly packed together in the basal ganglia, and the shape of these structures is highly irregular and non-spherical. Thus, if using a standard spherical searchlight procedure in the striatum, there is a substantial risk of the searchlight decoding from voxels positioned across anatomical boundaries, for which the interpretation would be difficult. That said, a searchlight is arguably more appropriate for cortical regions, where the risk of decoding across functional neuroanatomical boundaries is less severe (albeit still possible). In our ROI analysis of the striatum, we therefore relied on an a-priori functional parcellation of the striatum into five functional zones (Pauli et al., 2016)*”

I am left confused by the justification of the specific cross-subject cross-validation procedure in the rebuttal of R1’s comment #9. What are the implications of the observation that “results were unstable across repeated analysis runs, if we e.g. created 5 * 80/20 splits or 10 * 90/10 splits.” Should we be concerned about this?

When we mentioned unstable results in our response to the first reviewer’s previous comment, we were referring only to the non-significant results reported alongside our main striatal findings in Figure 3 b,c. As shown in the newly added supplementary figures 8 and 9, our main findings (correlation between identity classifier accuracy in the caudate nucleus and explicit knowledge; correlation between value classifier accuracy in the nucleus accumbens and subjective rating changes) reported in the same figure were indeed stable from the first cross-validation iteration.

To be clear, we developed this iterative cross-validation approach in order to mitigate the effects of researcher degrees of freedom which has been raised as a general concern about data analysis techniques used throughout the social and biological sciences, not because results were unstable. The approach we used is arguably less biased than traditional methods for dimensionality reduction used in the fMRI literature, because we are using a principled method to reduce the dimensionality of the data that doesn't depend on a subjective judgment on our part.

We have elaborated on the explanation of our approach in the Methods section (page 31, paragraph 3):

"*We followed an unbiased algorithmic approach to the selection of the regularization parameter C in the SVM and the number of retained components after PCA-based dimensionality reduction. In doing so we went beyond the current practice in the fMRI literature to either not report these hyperparameters at all, or to select them according to one of several possible heuristics. Here, we selected 80% (n=20) of participants for optimization of these hyperparameters, to then apply these hyperparameters during the analysis of the remaining 20% (n=5) of participants. To ensure that performance of a classifier for a given participant was not affected by which subset of participants was included in this parameter optimization, we repeated this procedure for 1000 iterations. We found that this approach resulted in a modal value of 16 for the number of retained PCA components, and a modal value of 1 for the regularization parameter C in the SVM. While the main findings were stable from the first iteration, the non-significant correlations appear less stable until a higher number of iterations (supplementary figures S7, S8). We speculate that if an area does not represent information of use for a classifier, our optimization approach would result in a noisy hyperparameter selection, resulting in noisy estimates of classifier performance when these parameters are applied in the analysis of the remaining participants, and will eventually result in non-significant findings as well. Overall, had we not followed this iterative process, and instead had used e.g. standard k-fold cross-validation or had made heuristic choices for these hyperparameters, we may have erroneously reported a false positive findings.*"

It is not so clear that there is not enough power to assess learning-related effects by comparing e.g. first vs second half of the trials per session. Please elaborate.

We reiterate that we optimized the paradigm from our 2015 study to test the specific experimental hypotheses described in the introduction of our manuscript. We do also agree with the reviewer that, for example, comparing the first and second half of the trials of each session can potentially assess learning-related effects. However, this would represent only one of several feasible post-hoc exploratory analyses. For example, it is possible that learning-related changes unfold at a different scale in the nucleus accumbens, the caudate nucleus, and the OFC. We believe that the present data set (as well as the 2015 study) represent an opportunity

for these exploratory analyses, which is the reason we chose to share both data and analysis scripts publicly.

We continue to be concerned about loss of statistical power when using only half the trials for a the MVPA analysis. We chose the number of trails in this study based on results of relevant studies from our lab, to test the experimental hypothesis described in this manuscript. Reducing the number of trials used for training and testing of a classifier will inevitably decrease classifier accuracy, which in turn reduces statistical power when comparing classifier accuracy to chance. The reduction in classifier accuracy with decreasing trials is plotted below and was created by running simulations analogous to the ones reported in the methods section of the manuscript. The manuscript parameters were used, except that we asked how classifier performance would be reduced by decreasing the number of trials for training and testing, even if every participant formed the correct outcome expectation in every trial. As can be seen there is a substantial reduction of classifier accuracy as one reduces the number of trials used in the training and test set.

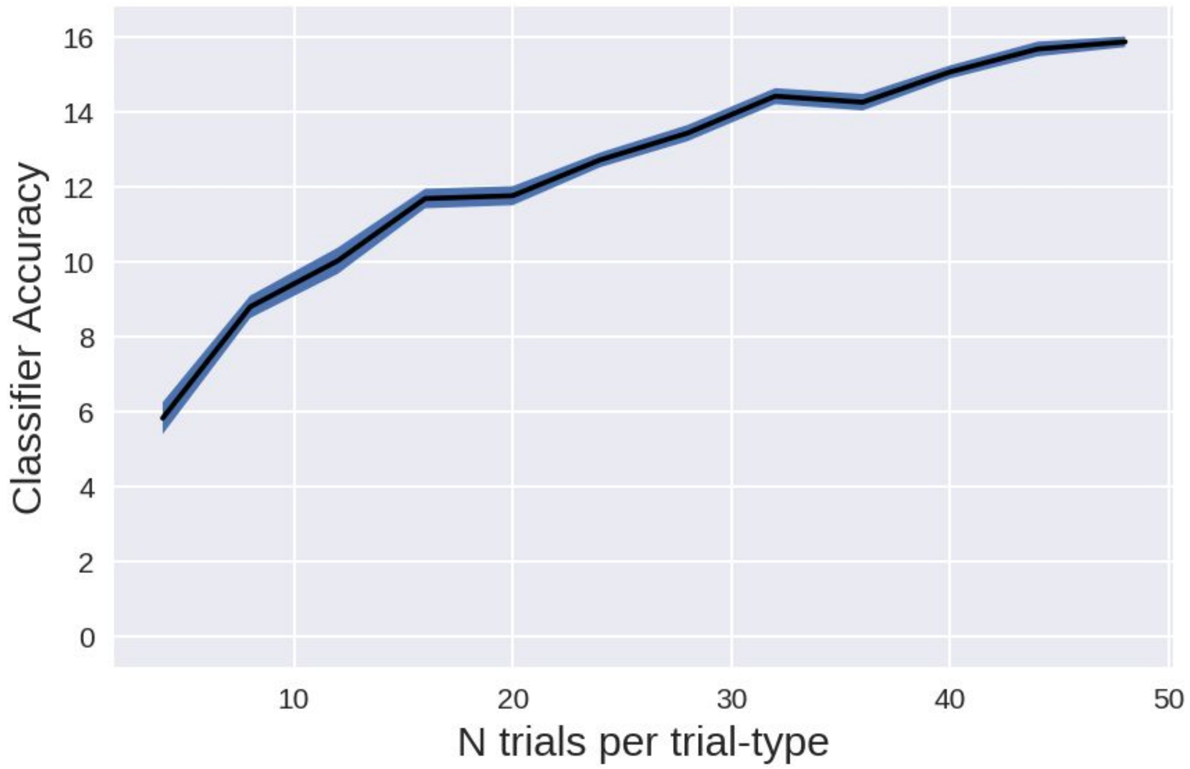

It is unclear how the cross-validation procedure, in which the classifier was trained on different sessions than the sessions on which it was tested, address the question whether the temporal nature of the task (i.e. presentation of distal and proximal CSs in close succession) introduced confound. The finding that there is little shared variance between the distal and proximal CS is reassuring, but nevertheless I would suggest to add this issue as a caveat that is hard to fully rule out in the discussion.

We added the following caveat to the discussion section (page 21, paragraph 3):

"A possible confound in this study is that when the classifier was trained on fitted BOLD responses to proximal conditioned stimuli and tested on fitted BOLD responses to the distal conditioned stimuli, decoding success could potentially be influenced by the distal stimulus eliciting a BOLD response that persists well past the proximal stimulus, rather than by similar, but statistically independent BOLD responses to distal and proximal stimuli. We attempted to rule out this possibility in our experimental design by ensuring that the experimentally induced jitter between the two stimulus events effectively decorrelated their associated hemodynamic responses, and indeed we confirmed that the correlation between modeled canonical hemodynamic responses at these two time points was very low. However, we cannot completely exclude the possibility that subtle temporal auto-correlations could contribute to the results."

Are the data presented here the same as those presented in your 2015 paper?

No, the data are completely new and were run in a new set of participants. There is no overlap with the dataset reported in the 2015 paper. The present study was indeed inspired by the study described in our 2015 paper, but optimized for the aims of the present study.

The manuscript already mentioned in the introduction and methods section that we optimized the paradigm from the 2015 study for the aims of the present study, but we appreciate the reviewer's comment and added the following lines to list the differences between the present and the previous 2015 study (page 23, paragraph 2):

"The present study was inspired by the experimental design used in a previous study (Pauli et al. 2015), but the experimental design used in the present study was optimized for the aims of the present study: (1) the previous study included aversive trial outcomes (unconditioned stimuli), which were not the focus of the present study, (2) we reduced the number of incongruent trials compared to the previous study (because we were interested in decoding neural representation of conditioned stimuli, rather than finding reward prediction errors in incongruent trials), and above all (3) the experimental contingencies were designed such that we could train/test machine learning classifiers on two orthogonal classification tasks: classification based on stimulus identity vs. based on stimulus value."

I generally like to see the actual changes implemented in the ms reiterated in the rebuttal letter. For next time.

Thank you for this suggestion - we have now done this in the present reply.

Reviewers' Comments:

Reviewer #3:
Remarks to the Author:
Thank you, I have no further comments.
